# 8-Oxoguanine DNA glycosylase 1 selectively modulates ROS-responsive NF-κB targets through recruitment of MSK1 and phosphorylation of RelA/p65 at Ser276

**DOI:** 10.1016/j.jbc.2023.105308

**Published:** 2023-09-29

**Authors:** Yaoyao Xue, Chunshuang Li, Shihua Deng, Xin Chen, Jinling Han, Xu Zheng, Miaomiao Tian, Wenjing Hao, Lang Pan, Istvan Boldogh, Xueqing Ba, Ruoxi Wang

**Affiliations:** 1 Key Laboratory of Molecular Epigenetics of Ministry of Education, Northeast Normal University, Changchun, Jilin, China; 2 College of Life Sciences, Northeast Normal University, Changchun, Jilin, China; 5 Institute of Biomedical Sciences, College of Life Sciences, Key Laboratory of Animal Resistance Biology of Shandong Province, Shandong Normal University, Jinan, Shandong, China; 3 Institute of Genetics and Developmental Biology, Chinese Academy of Sciences, Beijing, China; 4 Department of Microbiology and Immunology, University of Texas Medical Branch at Galveston, Galveston, USA

**Keywords:** ROS signaling, 8-oxoguanine, OGG1, MSK1, inflammatory genes

## Abstract

Nuclear factor kappa B (NF-κB) activity is regulated by various posttranslational modifications, of which Ser276 phosphorylation of RelA/p65 is particularly impacted by reactive oxygen species (ROS). This modification is responsible for selective upregulation of a subset of NF-κB targets; however, the precise mechanism remains elusive. ROS have the ability to modify cellular molecules including DNA. One of the most common oxidation products is 8-oxo-7,8-dihydroguanine (8-oxoGua), which is repaired by the 8-oxoguanine DNA glycosylase1 (OGG1)-initiated base excision repair pathway. Recently, a new function of OGG1 has been uncovered. OGG1 binds to 8-oxoGua, facilitating the occupancy of NF-κB at promoters and enhancing transcription of pro-inflammatory cytokines and chemokines. In the present study, we demonstrated that an interaction between DNA-bound OGG1 and mitogen-and stress-activated kinase 1 is crucial for RelA/p65 Ser276 phosphorylation. ROS scavenging or OGG1 depletion/inhibition hindered the interaction between mitogen-and stress-activated kinase 1 and RelA/p65, thereby decreasing the level of phospho-Ser276 and leading to significantly lowered expression of ROS-responsive cytokine/chemokine genes, but not that of *Nfkbis*. Blockade of OGG1 binding to DNA also prevented promoter recruitment of RelA/p65, Pol II, and p-RNAP II in a gene-specific manner. Collectively, the data presented offer new insights into how ROS signaling dictates NF-κB phosphorylation codes and how the promoter-situated substrate-bound OGG1 is exploited by aerobic mammalian cells for timely transcriptional activation of ROS-responsive genes.

The level of reactive oxygen species (ROS) significantly surpassing the capacity of antioxidant defenses leads to oxidative stress. It has been widely acknowledged that only the indiscriminate and stochastic ROS are toxic, whereas the compartmentalized and regulated ROS have been characterized as signaling molecules (1). ROS may damage DNA, and 8-oxo-7,8-dihydroguanine (8-oxoGua) is the most abundant oxidized product in the genome (2). It is recognized and excised by 8-oxoguanine DNA glycosylase1 (OGG1) and the resulting abasic site is repaired through the base excision repair pathway (3, 4). However, increasing evidence indicates that when 8-oxoGua is located at regulatory regions and bound by its cognate protein OGG1, it may function as an epigenetic-like mark in the transcriptional regulation of ROS-responsive genes (5, 6). Under oxidative stress, OGG1’s excision activity is lost, but it still retains the binding ability with the substrate. Thereafter, OGG1 alters the local architecture of DNA, recruits transacting factors such as nuclear factor kappa B (NF-κB) to the adjacent binding site to facilitate the assembly of transcriptional machinery, and consequently upregulates transcription from a set of NF-κB target genes (7, 8, 9).

While numerous studies have elucidated the regulatory mechanisms of NF-κB, the implication of guanine oxidation and OGG1 recognition in gene expression regulation presents an unexpected and brand-new perspective on that how NF-κB activity is controlled. NF-κB is a pivotal transcription factor that is activated to induce different genes in response to various extracellular signals. It is well acknowledged that the phosphorylation and the subsequent ubiquitination-mediated degradation of the inhibitory subunit IκB result in the liberation of NF-κB dimer and its translocation to the nucleus (10, 11). However, this step is necessary, but not sufficient, for the activation of NF-κB–dependent target genes (12, 13). Posttranslational modifications, such as phosphorylation, ubiquitination, acetylation, methylation, nitrosylation, and glycosylation primarily of RelA/p65 subunit, impose an additional layer of complexity to the modulation of its activity. Among the various posttranslational modifications, phosphorylation of RelA/p65 at serine 276 (S276) is of particular interest (14, 15) because it regulates NF-κB transcriptional activity in a gene-specific manner (16, 17). However, the precise molecular mechanism has not yet been elucidated.

We propose that under oxidative stress, OGG1 not only facilitates occupancy of NF-κB at the binding site but also serves as a bridge for the kinase to phosphorylate RelA/p65. Data of the present study showed that inflammatory stimulation enhanced the interactions among DNA-bound OGG1, mitogen-and stress-activated kinase 1 (MSK1), and RelA/p65, which are necessary for RelA/p65 phosphorylation. Depletion or inhibition of OGG1 did not prevent RelA/p65 nuclear translocation but did impede its interactions with MSK1, thereby decreasing levels of phospho-Ser276 of RelA/p65. Taken together, these data suggested a novel regulatory mechanism, by which OGG1 executes selective control over NF-κB–dependent inflammatory genes.

## Results

### OGG1 selectively regulates the expression of ROS signaling–dependent and NF-κB–driven pro-inflammatory cytokines/chemokines

**Figure 1:**
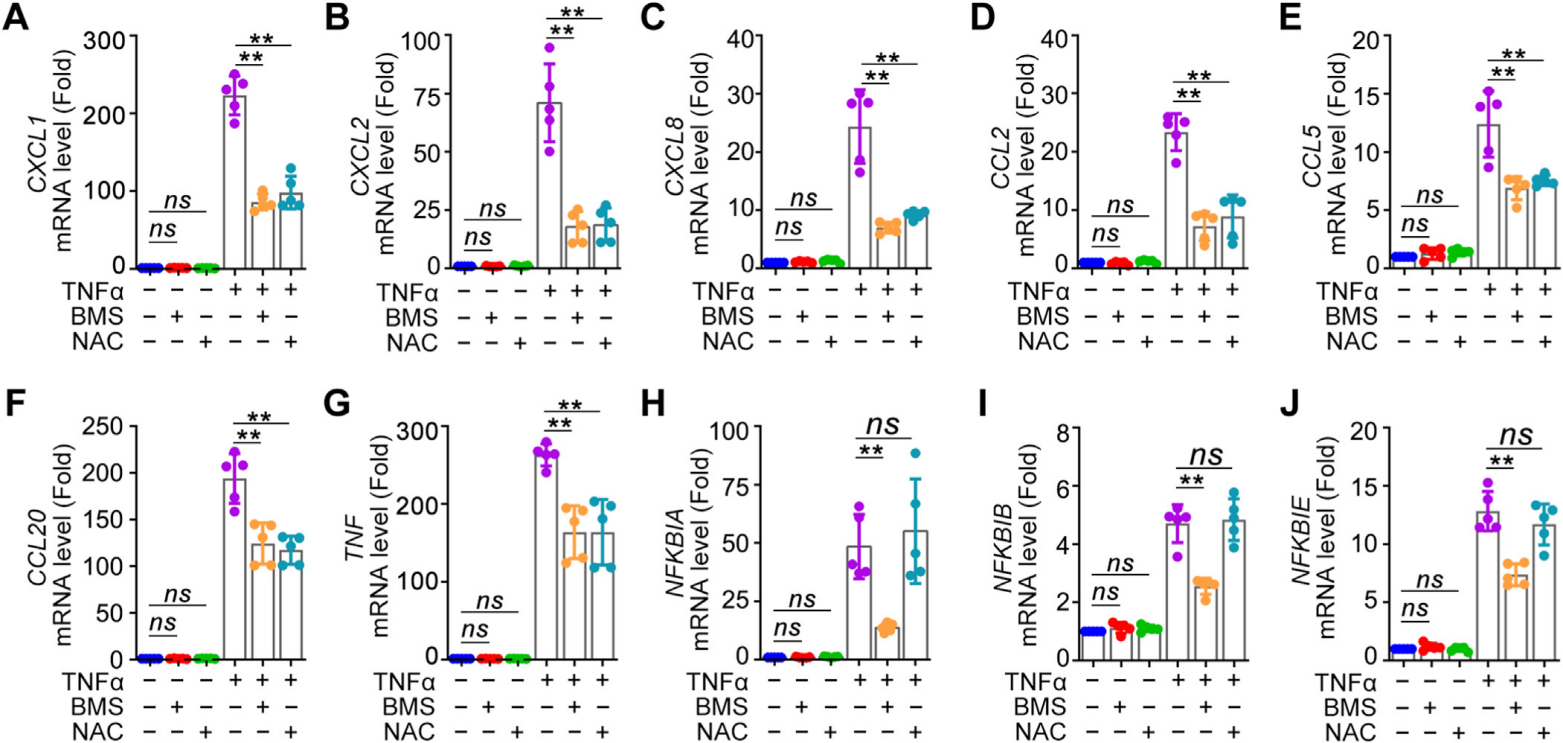
**ROS selectively regulates NF-κB–driven inflammatory gene expression**. *A*–*J*, HEK293 cells were exposed to TNFα (20 ng/ml) for 1 h with or without pretreatment of BMS-345541 (10 μM) or NAC (10 mM). *CXCL1*, *CXCL2*, *CXCL8*, *CCL2*, *CCL5*, *CCL20*, *TNF*, *NFKBIA*, *NFKBIB*, and *NFKBIE* gene expression was measured by real-time qPCR. All experiments were performed five times. Data are presented as mean ± SD. ∗∗*p* < 0.01, ns, not significant. NAC, N-acetyl-L-cysteine; qPCR, quantitative PCR; ROS, reactive oxygen species.

To verify our hypothesis, HEK293 cells were exposed to tumor necrosis factor (TNF)α, and the expression of inflammatory genes (*CXCL1*, *CXCL2*, *CXCL8*, *TNF*, *CCL2*, *CCL5*, *CCL20, NFKBIA*, *NFKBIB*, and *NFKBIE*) was determined by quantitative PCR (qPCR). TNFα stimulation induced a rapid upregulation of these genes, with a significant elevation observed at 1 h (Fig. S1*A*). Similar results were observed in MEF cells upon TNFα stimulation (Fig. S1*B*). To validate that NF-κB regulates the expression of these inflammatory genes, cells were treated with the IKK inhibitor BMS-345541 1 h prior to TNFα administration (18). The result showed that treatment of 10 μM BMS-345541 almost completely abrogated TNFα-induced upregulation of the inflammatory genes (Fig. 1, *A*–*J*).

To investigate whether ROS is involved in the transcription of NF-κB–driven genes, cells were treated with or without TNFα in the presence or absence of 10 mM GSH precursor N-acetyl-L-cysteine (NAC) (19) and then gene expression was measured. Based on whether their expression was affected by ROS scavenging, the tested genes were subdivided into two groups. The first group (group I) includes 7 genes (*CXCL1*, *CXCL2*, *CXCL8*, *CCL2*, *CCL5*, *CCL20*, *TNF*), and the expression of these genes was significantly induced by TNFα, which, however, was markedly mitigated by NAC administration (Fig. 1, *A*–*G*). The second group (group Ⅱ) includes 3 genes (*NFKBIA*, *NFKBIB*, and *NFKBIE*), whose expression was not affected by NAC (Fig. 1, *H*–*J*).

**Figure 2:**
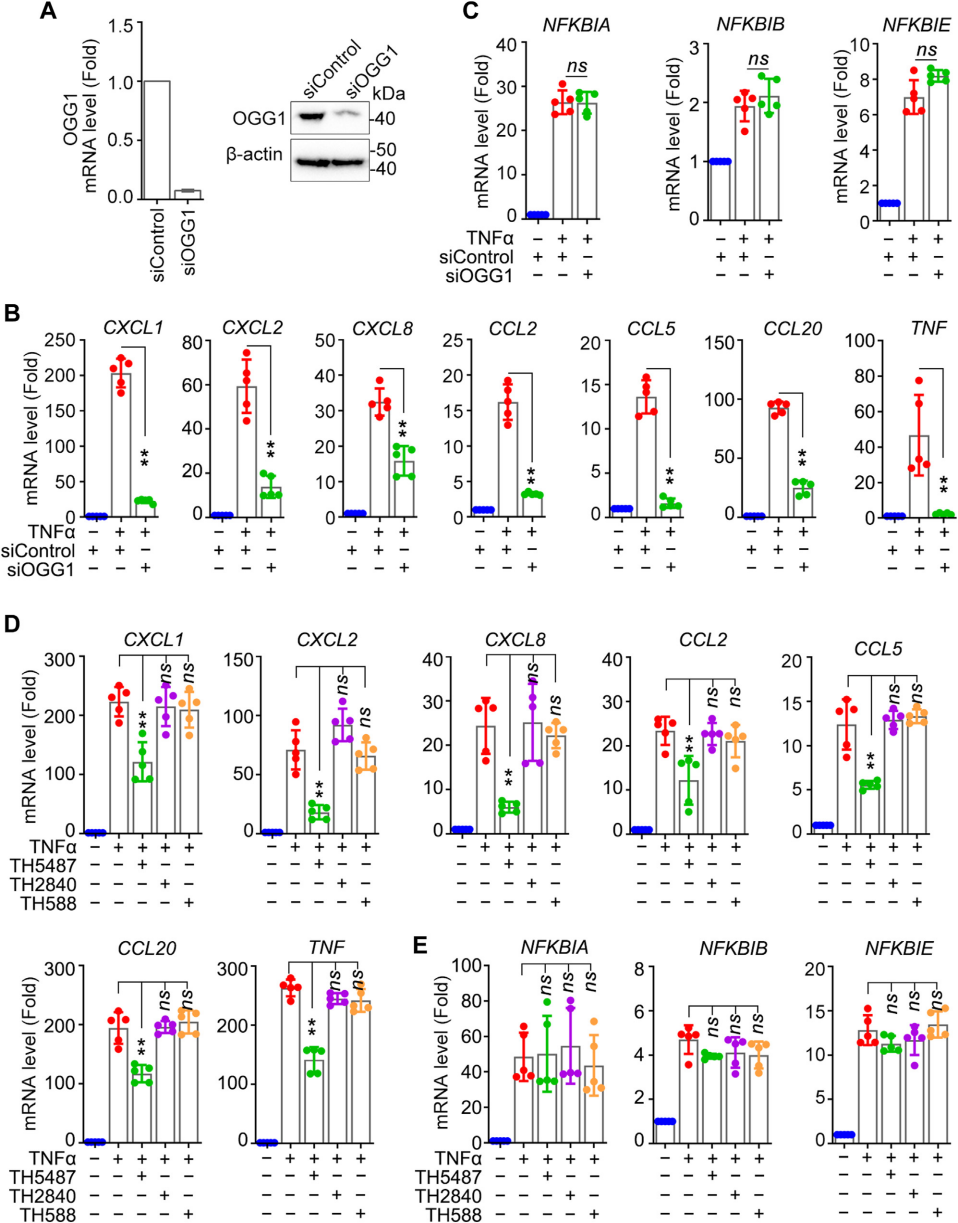
**OGG1 selectively regulates ROS signaling–dependent NF-κB–driven gene expression.***A*, verification of OGG1 interference efficacy. HEK293 cells were transfected with siRNA targeting OGG1 or control for 48 h, followed by TNFα incubation for 1 h. OGG1 expression was assessed through Western blotting and qPCR in in HEK293 cells. *B* and *C*, effects of OGG1 knockdown on NF-κB–driven gene expression. HEK293 cells were transfected with siRNA targeting OGG1 and exposed to TNFα for 1 h. The mRNA levels of *CXCL1*, *CXCL2*, *CXCL8*, *CCL2*, *CCL5*, *CCL20*, *TNF* (B) and *NFKBIA*, *NFKBIB*, *NFKBIE* (C) gene were assessed by qPCR. *D* and *E*, effects of OGG1 inhibitors on NF-κB–driven gene expression. HEK293 cells were exposed to TNFα for 1 h with or without pretreatment of TH5487, TH2840, or TH588. *CXCL1*, *CXCL2*, *CXCL8*, *CCL2*, *CCL5*, *CCL20*, *TNF* (*D*) and *NFKBIA*, *NFKBIB*, *NFKBIE* (*E*) gene expression was measured by qPCR. All experiments were performed five times. Data are expressed as mean ± SD. ∗∗*p* < 0.01, ns, not significant. OGG1, 8-oxoguanine DNA glycosylase 1; qPCR, quantitative PCR; ROS, reactive oxygen species.

Our previous studies revealed that under oxidative stress, OGG1 is immobilized to DNA through 8-oxoGua and promotes the DNA occupancy of NF-κB, thus enhancing the transcription of pro-inflammatory genes (7, 8, 9). To further specify the role of OGG1 in NF-κB–dependent gene expression, HEK293 cells were transfected with siRNA against *OGG1 (siOGG1)* or control and then exposed to TNFα. The expression of OGG1 mRNA and protein was decreased by ∼80% in *siOGG1*-treated cells, confirming acceptable interference efficacy (Fig. 2*A*). The qPCR results indicated that OGG1 silencing significantly inhibited TNFα-induced expression of group I genes but did not affect that of group Ⅱ genes (Fig. 2, *B* and *C*). The data suggested that OGG1 selectively regulates ROS signaling–dependent NF-κB–driven gene expression.

To further verify this specificity of OGG1, the small molecule OGG1 inhibitor TH5487 was utilized. TH5487 does not alter the expression of OGG1 at the RNA or protein level during the experimental time frame but prevents OGG1 from binding to 8-oxoGua-containing DNA (20). Similar to OGG1 interference, treatment of TH5487 significantly decreased mRNA levels of group Ⅰ genes without affecting that of group Ⅱ genes (Fig. 2, *D* and *E*). TH2840, an inactive analog of TH5487 (20), had no effect on gene expression of both group I and group II genes. TH588, an inhibitor of 7,8-Dihydro-8-oxoguaninetriphosphatase (MTH1) that eliminates 8-oxo-7,8-dihydro-2′-deoxyguanosine triphosphate through its nucleoside triphosphate pyrophosphatase activity (21), also had no effect on TNFα-stimulated gene expression (Fig. 2, *D* and *E*). Similar to DMSO, TH5487, TH2840, or TH588 had no effect on gene expression without TNFα stimulation (Fig. S2*A*), implying the specificity of OGG1 in gene transcription regulation under oxidative stress. The same results were obtained when BMS-345541, NAC, or TH5487 were applied to MEF cells (Fig. S2*B*). Parallel cultures of WT (*Ogg1*^+/+^) and *Ogg1* KO (*Ogg1*^−/−^) MEF cells were stimulated by TNFα, and gene expression was determined. Results showed that mRNA levels of group I genes in *Ogg1* KO cells were significantly lower than WT cells but that of group II genes showed no difference (Fig. S2*C*). Taken together, these data indicated that OGG1 is required for transcription activation of a subgroup of ROS-responsive NF-κB targets.

### RelA/p65 S276 phosphorylation is responsive to ROS and selectively necessary for the expression of pro-inflammatory cytokines/chemokines

**Figure 3:**
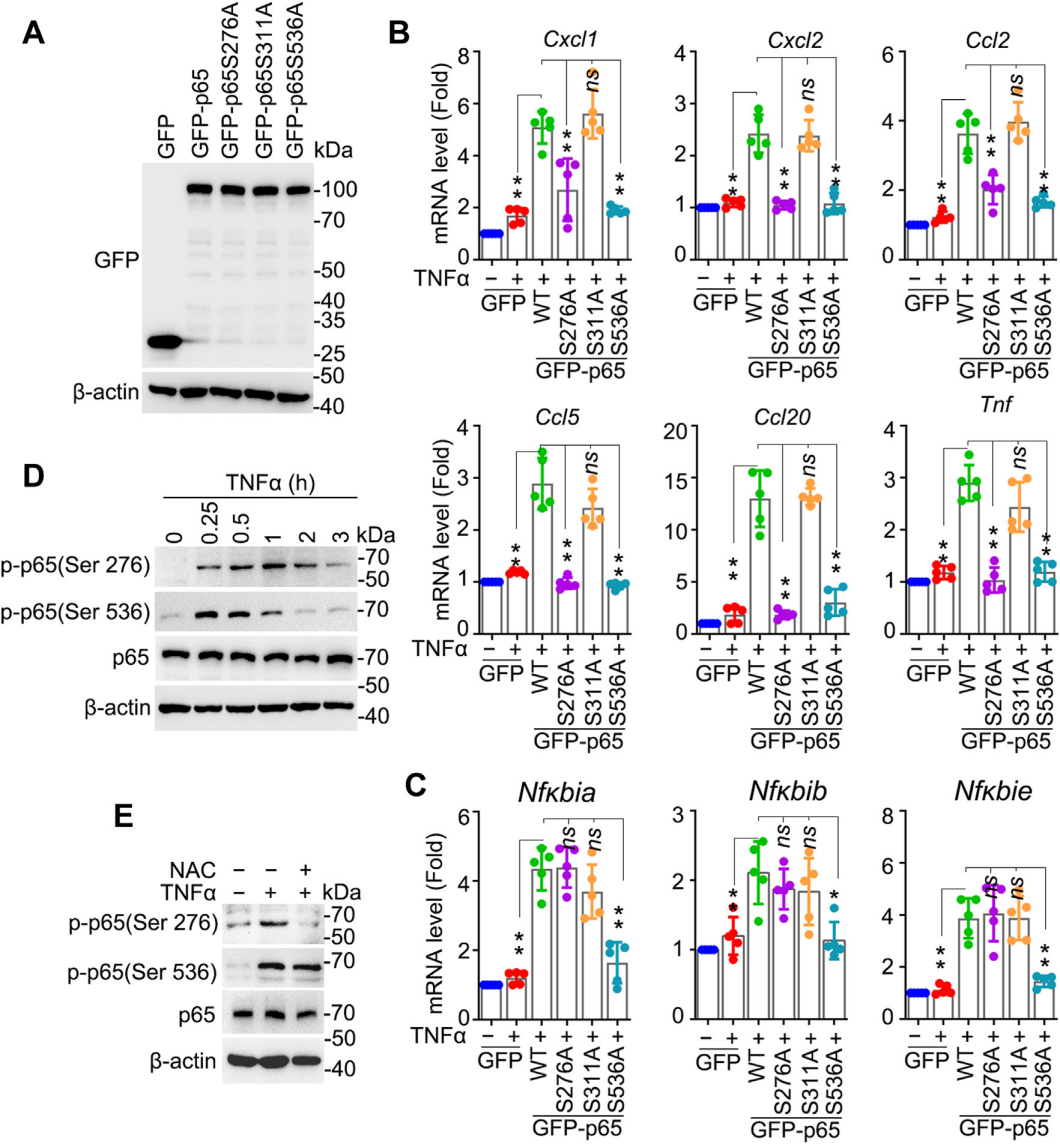
**ROS signaling–responsive RelA/p65 Ser276 phosphorylation is essential for pro-inflammatory cytokines/chemokines expression.***A*–*C*, RelA/p65 Ser276 phosphorylation is crucial for pro-inflammatory cytokines/chemokines expression. *RelA/p65*^*−/−*^ MEF cells were transfected with plasmids expressing GFP-tagged wt RelA/p65 or site-specific mutants and then exposed to TNFα for 1 h. Western blotting confirmed the comparable expression levels of GFP-tagged wt RelA/p65 and site-specific mutants (*A*). *Cxcl1*, *Cxcl2*, *Ccl2*, *Ccl5*, *Ccl20*, *Tnf*, *Nfkbia*, *Nfkbib*, and *Nfkbie* gene were assessed by qPCR (*B* and *C*). *D*, kinetic changes in the phosphorylation of RelA/p65 at Ser276. HEK293 cells were exposed to TNFα for varying durations (0, 0.25, 0.5, 1, 2, 3 h). Western blot analysis was performed to detect the phosphorylation of RelA/p65 at Ser276 and Ser536. *E*, RelA/p65 Ser276 phosphorylation responds to ROS Signaling. HEK293 cells were exposed to TNFα for 1 h with or without pretreatment of NAC. Phosphorylation of RelA/p65 at Ser276 and Ser536 was detected by Western blot. All experiments were performed five times. Data are expressed as mean ± SD. ∗*p* < 0.05, ∗∗*p* < 0.01, ns, not significant. NAC, N-acetyl-L-cysteine; qPCR, quantitative PCR; ROS, reactive oxygen species.

Phosphorylation of RelA/p65 at serine(s) 276, 311, 536, or other sites constitutes a crucial layer for regulation of NF-κB activation following TNFα stimulation (17). Among these phosphorylation events, it was determined that RelA/p65 Ser276 phosphorylation is selectively controlled by ROS signaling (16). In this study, we investigated the dependence of RelA/p65 phosphorylation at these serine sites for NF-κB–driven gene expression. Human GFP-RELA/p65 and mutant GFP-RELA/p65 S276A, S311A, S536A plasmids were constructed and transfected in *RelA*/*p65*^*−/−*^ MEF cells. Equal expressions of the exogenous GFP-RELA/p65 and the mutants were verified by western blotting (Fig. 3*A*). To examine the impact of phosphorylation of RelA/p65 at different serine sites, RNA was prepared from resting and TNFα-treated MEF cells, and the expression of ten NF-κB–driven genes was analyzed. Results which showed the transcriptional activity of RelA/p65 S276A was markedly impaired, which inhibited the expression of group Ⅰ genes, but not that of group Ⅱ genes. The transcriptional activity of RelA/p65 S536A was completely suppressed with both group Ⅰ and group II genes, whereas RelA/p65 S311A mutation did not impact the expression of any tested gene (Fig. 3, *B* and *C*). These results suggested that RelA/p65 Ser276 phosphorylation selectively regulates those genes dependent on ROS signaling.

Next, we investigated the impact of ROS signaling on RelA/p65 phosphorylation. TNFα induced a rapid phosphorylation of Ser276 RelA/p65, peaking at 30 min to 1 h, and rapidly returning to the basal level 2 h after stimulation (Fig. 3*D*), which aligns with the trend in gene expression (Fig. S1*A*). In contrast, phospho-Ser536 RelA/p65 reached its peak value 15 min after induction (Fig. 3*D*). Nevertheless, we observed that the high level of TNFα-induced phospho-Ser276 of RelA/p65 was completely abrogated after NAC treatment, whereas the induction of phospho-Ser536 of RelA/p65 was not affected (Fig. 3*E*), consistent with previous studies (12, 22). Additionally, NAC had no effect on phospho-Ser276 RelA/p65 without TNFα stimulation (Fig. S3*A*). Similar results were obtained using MEF cells (Fig. S3, *A*–*C*).

### OGG1 is crucial for the phosphorylation of RelA/p65 at Ser276

**Figure 4:**
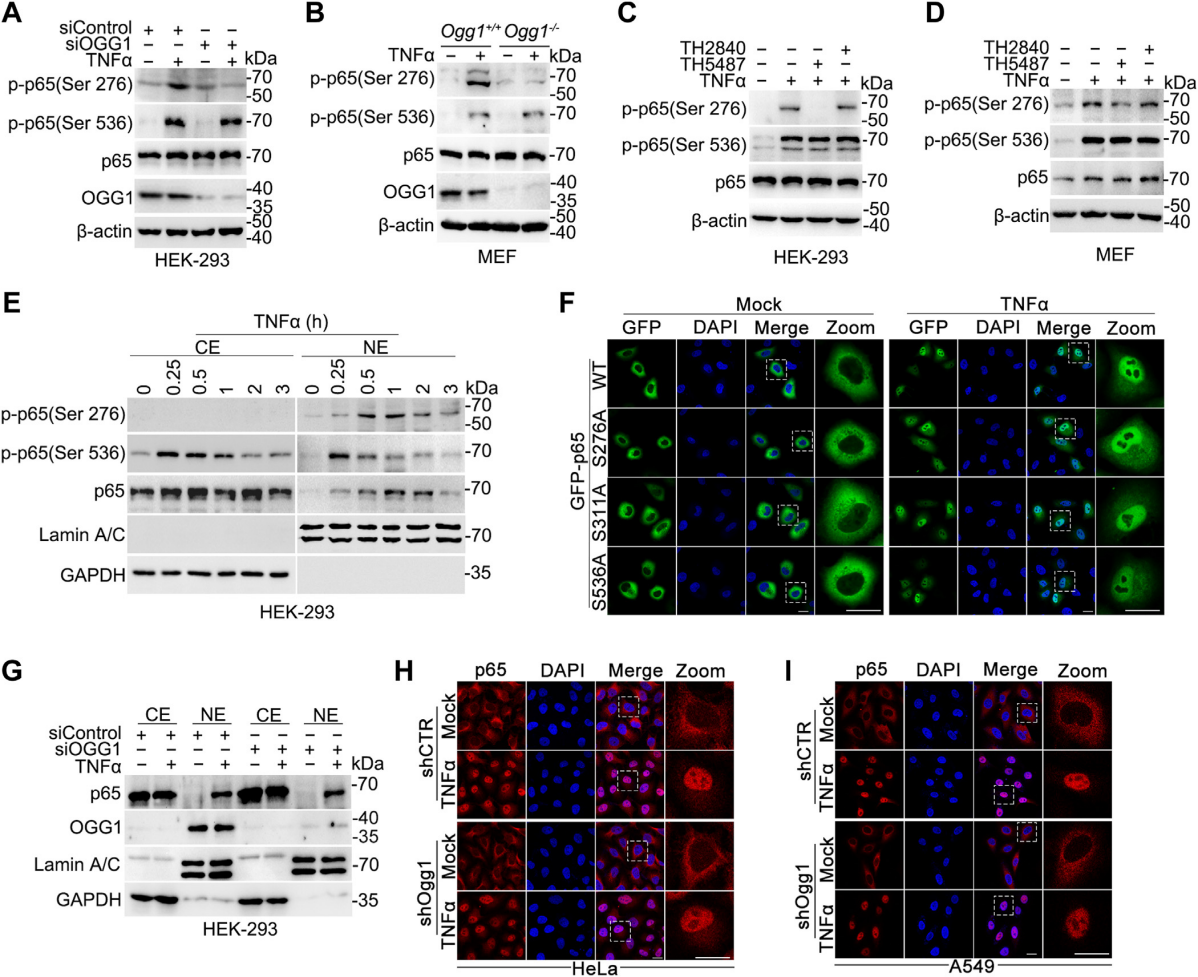
**OGG1 is required for Ser276 phosphorylation of RelA/p65 but not for its nuclear translocation.***A*–*D*, OGG1 is required for phosphorylation of RelA/p65 at Ser276. HEK293 cells were transfected with siRNA targeting OGG1 and then exposed to TNFα for 1 h (*A*); *Ogg1*^*+/+*^ or *Ogg1*^*−/−*^ MEF cells were treated with TNFα for 1 h (*B*); HEK293 cells (*C*) and *Ogg1*^*+/+*^ or *Ogg1*^*−/−*^MEF cells (*D*) were exposed to TNFα for 1 h with or without pretreatment of TH5487 or TH2840; and then RelA/p65 Ser276 phosphorylation and Ser536 phosphorylation were detected by Western blot. *E*, RelA/p65 is phosphorylated in the nucleus compartment. HEK293 cells were exposed to TNFα for varying durations (0, 0.25, 0.5, 1, 2, 3 h) and then fractionated into cytoplasmic extraction (CE) and nuclear extraction (NE) preparations for Western blotting using the indicated Abs. *F*, RelA/p65 nuclear translocation is independent of Ser276 phosphorylation. HeLa cells were transfected with plasmids expressing GFP-tagged wt RelA/p65 or site-specific mutants and exposed to TNFα for 30 min. Subcellular localization of wt RelA/p65 or site-specific mutants was detected by immunofluorescence. The nuclei were counter stained with DAPI (*blue*: DNA; *green*: GFP). Scale bar represents 20 μm. *G*–*I*, OGG1 has no role in translocation induction of RelA/p65. HEK293 cells were transfected with siRNA targeting OGG1 or control for 48 h and then incubated with TNFα for 30 min. Cells were fractionated into cytoplasmic extraction (CE) and nuclear extraction (NE) preparations for analysis by Western blotting using the indicated Abs (*G*). HeLa-shCTR or HeLa-shOGG1 cells (*G*) and A549-shCTR or A549-shOGG1 (*I*) were exposed to TNFα for 30 min. Subcellular localization of RelA/p65 were detected by immunofluorescence. The nuclei of cells were counter stained with DAPI (*blue*: DNA; *red*: RelA/p65). Scale bar represents 20 μm. A representative result of three experiments is shown. OGG1, 8-oxoguanine DNA glycosylase 1.

Given that OGG1 affects the expression of group Ⅰ genes but not that of group II genes (Figs. 2, *B*–*E* and S2, *B* and *C*), we questioned whether OGG1 impacts RelA/p65 phosphorylation at Ser276. HEK293 cells were transfected with siRNA targeting *OGG1*. Knockdown of OGG1 significantly decreased TNFα-induced phosphorylation of RelA/p65 at S276, whereas phosphorylation of RelA/p65 at S536 remained unchanged (Fig. 4*A*). Meanwhile, similar results were obtained from *Ogg1*^*+/+*^ and *Ogg1*^*−/−*^ MEF cells (Fig. 4*B*). Moreover, we tested the phosphorylation of RelA/p65 upon OGG1 inhibition. Likewise, TH5487 decreased the level of phospho-Ser276, but not phospho-Ser536 of RelA/p65 in HEK293 and MEF cells, while TH2840 did not affect either (Fig. 4, *C* and *D*). All inhibitors had no effect on the level of phospho-Ser276 of RelA/p65 without TNFα stimulation (Fig. S3*A*), implying the specificity of OGG1 enhancing the phospho-Ser276 modification of RelA/p65 under oxidative stress.

Next, we questioned the cellular location where RelA/p65 Ser276 phosphorylation occurs. Cells were exposed to TNFα, and cytoplasmic and sucrose cushion-purified nuclear extracts were obtained. While RelA/p65 phospho-Ser536 was observed in both the cytoplasm and nucleus, RelA/p65 phospho-Ser276 was exclusively present in the nuclear fractions (Fig. 4*E*). The kinetics of nuclear accumulation was in parallel with the level of phospho-Ser276 in whole cell lysate. To further determine whether the nuclear translocation of RelA/p65 depends on phosphorylation of serine at S276, we examined the nuclear translocation of RelA/p65 mutants with mutation at residue 276, 311, or 536 (S276A, S311A, or S536A). HeLa cells were transfected with GFP-RelA/p65-or mutant-expressing plasmids, then stimulated with TNFα for 30 min, and fluorescence microscopy was performed. The result showed that TNFα stimulation induced a marked nuclear import of GFP-RelA/p65; however, RelA/p65 nuclear translocation did not depend on its phosphorylation at site Ser276, Ser311, or Ser536 (Fig. 4*F*).

To determine whether OGG1 affects the nuclear translocation of RelA/p65, we used siRNA to knockdown OGG1 and detected the nuclear translocation of RelA/p65 by Western blot. The result showed that OGG1 knockdown did not affect the translocation of RelA/p65 into the nucleus in HEK293 cells (Fig. 4*G*). Additionally, we established HeLa and A549 cells with stable OGG1 silencing (Fig. S3*D*) and stimulated these cells with TNFα for 30 min. Immunofluorescence labeling showed that the presence or absence of OGG1 did not affect the nuclear translocation of RelA/p65 (Fig. 4, *H* and *I*). To further verify the effect of OGG1 on the nuclear translocation of RelA/p65 under TNFα stimulation, cytoplasmic and nuclear extracts were prepared from TNFα-stimulated *Ogg1*^*+/+*^ and *Ogg1*^*−/−*^ MEF cells. Western blotting showed that the knockout of OGG1 did not affect the time dynamics of RelA/p65 translocation into the nucleus (Fig. S3*E*). Taken together, these data suggested that after nuclear translocation, RelA/p65 requires OGG1 to facilitate its phosphorylation at S276, as well as its selective regulation of gene expression.

To signify the implication of oxidative stress in the effect of OGG1 on NF-κB phosphorylation and gene transcription, HEK293 cells were exposed to 200 μM H_2_O_2_ for 1 h, a concentration known to induce cellular oxidative stress (23). The phosphorylation and cellular localization of RelA/p65 were detected by Western blotting. The results showed that H_2_O_2_ alone could not induce phosphorylation at Ser276 or Ser536 of RelA/p65, nor RelA/p65 translocation into the nucleus (Fig. S4, *A* and *B*). Consequently, gene expression was not upregulated (Fig. S4*C*). Similar results were obtained by using MEF cells (Fig. S4, *B*–*F*). These finding suggested that oxidative modification of DNA, without inflammatory stimuli–induced NF-κB activation, is insufficient for OGG1 to modulate phospho-Ser276 of RelA/p65 and selectively regulate NF-κB–dependent gene transcription.

### Inflammatory stimulation induces the interaction between OGG1 and MSK1

**Figure 5:**
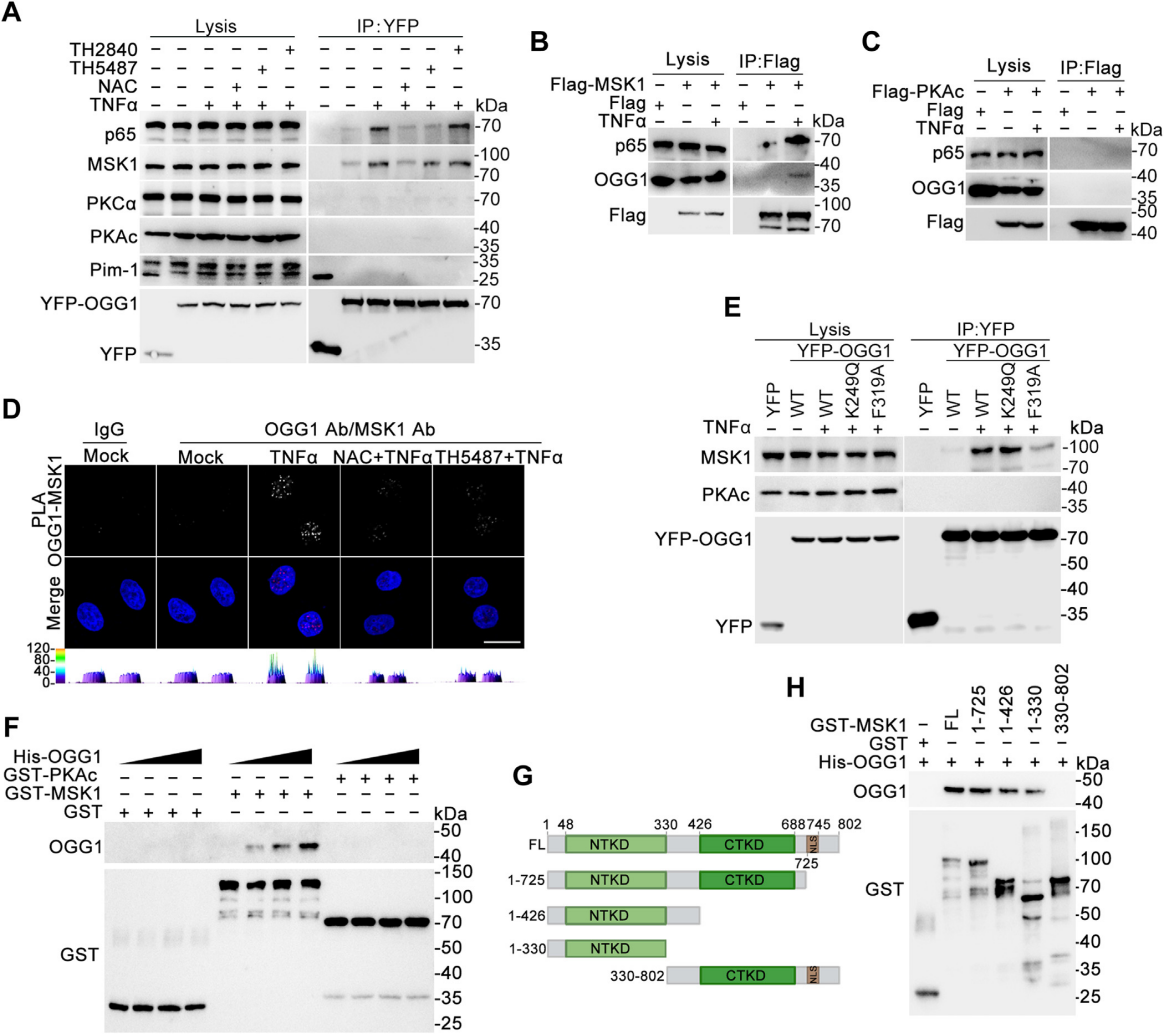
**TNFα stimulation induces ROS-responsive interaction of MSK1 with OGG1.***A*–*D*, TNFα stimulation induces binding of OGG1 to MSK1 in cells. HEK293 cells expressing YFP or YFP-OGG1 were subjected to mock treatment or TNFα exposure for 1 h in the presence or absence of 1 h pretreatment with NAC, TH5487, or TH2840. Whole-cell extracts were prepared, and immunoprecipitation was performed with GFP beads. Levels of MSK1, PKAc, Pim1, PKCα, and RelA/p65 were determined by Western blot (*A*). HEK293 cells expressing Flag-MSK1 or Flag-PKAc were treated with TNFα for 1 h. Whole-cell extracts were prepared, and immunoprecipitation was performed with Flag beads. Levels of OGG1 and RelA/p65 were determined by Western blot (*B* and *C*). HeLa cells were exposed to TNFα for 1 h. PLA was performed using OGG1 and MSK1 Abs. Cell nuclei were counter stained with DAPI. Scale bar represents 20 μm (*D*). *E*, OGG1 interaction with MSK1 independent of its base excision activity. HEK293 cells expressing YFP, YFP-OGG1, YFP-OGG1 K249Q, or YFP-OGG1 F319A were subjected to mock treatment or TNFα exposureto for 1 h. Whole cell extracts were prepared, and immunoprecipitation was performed with GFP beads. Levels of MSK1 and PKAc were determined by Western blot. *F*, physical interaction between OGG1 and MSK1 *in vitro*. Increasing amounts of His-OGG1 were incubated with GST, GST-MSK1, or GST-PKAc, and a pull-down assay was conducted. Levels of pulled-down OGG1 were detected by Western blot. *G*, schematic illustration of plasmids expressing full-length MSK1 or mutants. Schematics of the full-length (FL) GST-MSK1 as well as truncated domains and deletion mutants are shown. *H*, NTKD mediates the association of OGG1 with MSK1. His-OGG1 was incubated with GST-MSK1 or mutants, and a pull-down assay was performed. Levels of pulled-down OGG1 were detected by Western blot. A representative result of three independent experiments is presented. All experiments were performed three times. Data are expressed as mean ± SD. OGG1, 8-oxoguanine DNA glycosylase 1; MSK1, mitogen-and stress-activated kinase 1; NAC, N-acetyl-L-cysteine; NTKD, N-terminal kinase domain; PLA, proximity ligation assay; ROS, reactive oxygen species.

Because OGG1 lacks kinase activity, we sought to understand how OGG1 may play a role in the phosphorylation of transcription factors. We hypothesized that OGG1 might interact with a specific protein kinase and recruit it to phosphorylate RelA/p65 under oxidative stress. Previous studies have shown that the phosphorylation of RelA/p65 at S276 can be mediated by various kinases, including PKAc, MSK1/2, Pim1, PKCα, and RSK p90, in a stimulus-dependent manner (14). Notably, MSK1 activation is closely associated with ROS signaling (24, 25). To test our hypothesis, HEK293 cells were transfected with YFP-OGG1–expressing plasmid. Following TNFα treatment, co-immunoprecipitation (Co-IP) assays were performed, and OGG1-interacting proteins were identified using specific antibodies (Abs) to MSK1, PKAc, PKCα, and Pim-1. The results showed that 1 h TNFα treatment resulted in the association of OGG1 with MSK1 (Fig. 5*A*). Conversely, treatment with NAC and TH5487, but not TH2840, inhibited this interaction. Furthermore, Flag-MSK1– or Flag-PKAc–expressing HEK293 cells were treated with TNFα, and co-IP assays were performed. The result showed that Flag-MSK1, but not Flag-PKAc, interacted with OGG1 (Fig. 5, *B* and *C*). To further confirm the interaction of OGG1 with MSK1, *in situ* proximity ligation assay (PLA) were performed. The microscopic results showed that TNFα stimulation induced the interaction of OGG1 with MSK1, which was in the nucleus, while treatment with NAC and TH5487 diminished this interaction (Fig. 5*D*). These data suggested that OGG1 interacts with MSK1 on DNA in the nucleus under oxidative stress.

Next, we examined whether OGG1 enzymatic activity was essential for its interaction with MSK1. HEK293 cells were transfected with plasmids expressing YFP-tagged wtOGG1 or enzymatically deficient OGG1 mutants. The lysine 249 mutant (K249Q) of OGG1 lacks base excision activity but retains the ability to recognize and bind 8-oxoGua in DNA. The phenylalanine 319 mutant (F319A) of OGG1 has low affinity for 8-oxoGua (26, 27, 28, 29). HEK293 cells that expressed YFP-wtOGG1 or the variants were stimulated with TNF-α for 1 h and Co-IP assay was conducted. Intriguingly, YFP-OGG1 F319A displayed a reduced interaction with MSK1, compared with YFP-wtOGG1 and YFP-OGG1 K249Q (Fig. 5*E*). These data suggested that OGG1 primarily serves as a scaffold or bridge at the promoter, rather than actively participating in base excision.

Finally, we investigated a potential physical interaction between recombinant OGG1 and MSK1. MSK1 and PKAc proteins were generated using PGEX4T-2 expression vector and purified. GST-pull-down assays revealed a concentration-dependent physical interaction between OGG1 and MSK1, while no interaction was observed with PKAc (Figs. 5*F* and S5*A*). MSK1 comprises three domains: N-terminal kinase domain, C-terminal kinase domain, and nuclear location sequence (30, 31). To elucidate which domain(s) of MSK1 interacts with OGG1, we designed and generated four truncated MSK1 mutants: GST-MSK1 1-725, GST-MSK1 1-426, GST-MSK1 1-330, and GST-MSK1 330-803 (Fig. 5*G*). While His-OGG1 could be pulled down by other truncated mutants, GST-MSK1 330-803 did not exhibit an interaction with His-OGG1 (Fig. 5*H*). Subsequently, we employed His-OGG1 to pull down GST-MSK1 and its mutants, yielding the result which was consistent with GST-OGG1 pull-down (Fig. S5*B*). Collectively, these results suggested that the physical interaction between OGG1 and MSK1 relies on the NTK domain of the latter.

### OGG1 facilitates MSK1-mediated phosphorylation of RelA/p65 at Ser276

**Figure 6:**
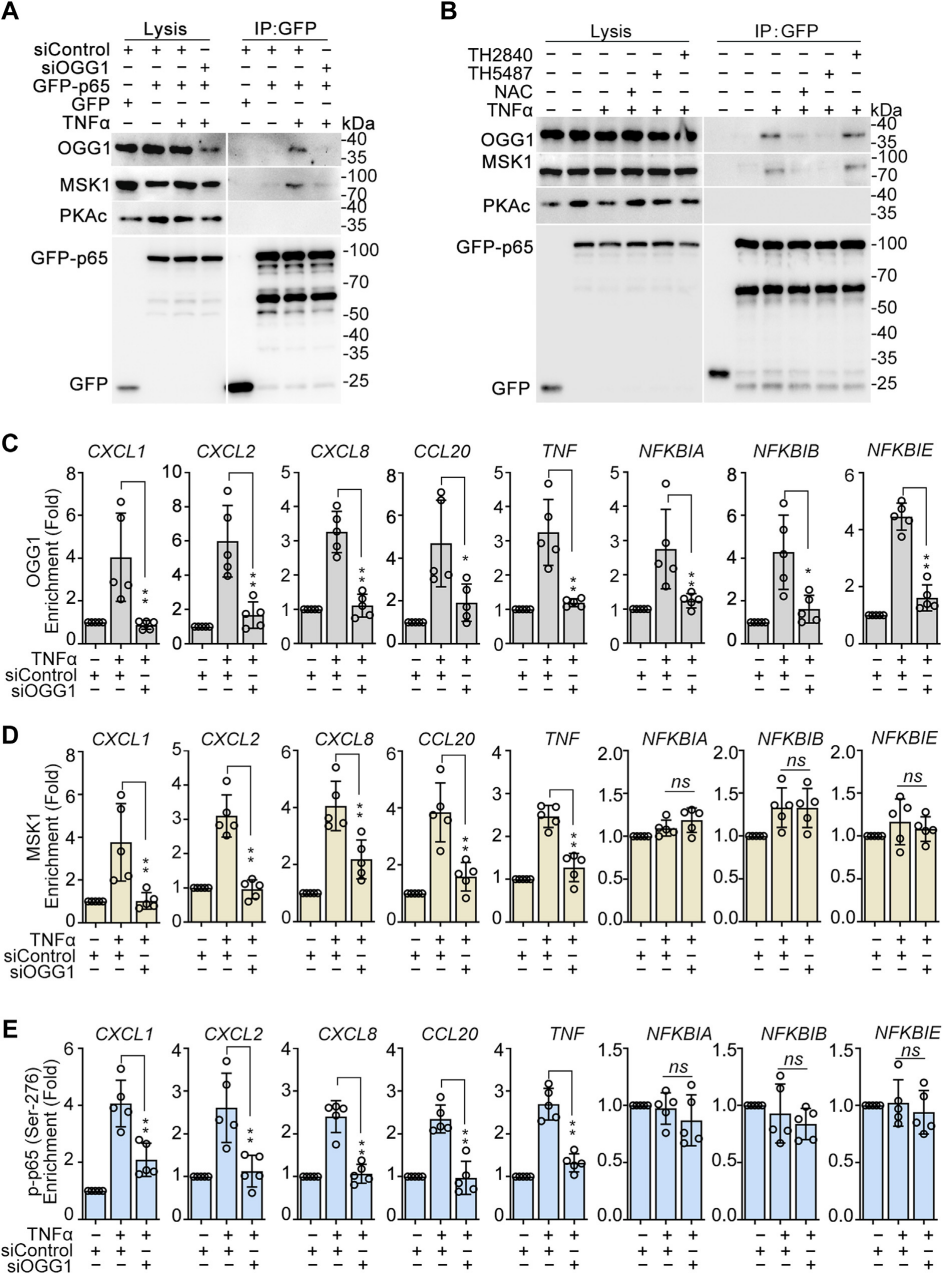
**OGG1 is crucial for MSK1-mediated phosphorylation of RelA/p65 at Ser276.***A* and *B*, OGG1 facilitates the interaction between p65 and MSK1 in cells. GFP or GFP-RelA/p65–expressing HEK293 cells were transfected with or without siRNA targeting OGG1 (*A*) or were mock-treated or treated with NAC, TH5487, TH2840, or not for 1 h (*B*) and then exposed to TNFα for 1 h. Whole cell extracts were prepared, and immunoprecipitation was performed with GFP beads. Levels of MSK1, PKAc, and OGG1 were determined by Western blot. *C*–*E*, OGG1 enhances MSK1 and p-RelA/p65 (Ser 276) enrichment in promoter regions of pro-inflammatory cytokine/chemokine genes. HEK293 cells were transfected with siRNA targeting OGG1 or control for 36 h and then incubated with TNFα for 1 h. Chromatin was isolated, and DNA was immunoprecipitated with OGG1 (*C*), MSK1 (*D*), and p-RelA/p65 (Ser 276) (*E*) antibodies. The results of ChIP analysis were determined by qPCR. Data are expressed as mean ± SD. ∗*p* < 0.05, ∗∗*p* < 0.01, ns, not significant. OGG1, 8-oxoguanine DNA glycosylase 1; MSK1, mitogen-and stress-activated kinase 1; NAC, N-acetyl-L-cysteine; qPCR, quantitative PCR.

To determine whether OGG1 recruits MSK1 to enhance the phosphorylation of S276 of RelA/p65, cells were transfected with siRNA targeting *OGG1* or a control, followed by incubation with TNFα. Co-IP results indicated a significant decrease in the interaction between RelA/p65 and MSK1 upon OGG1 knockdown (Fig. 6*A*). Additionally, pretreatment of NAC and TH5487, but not TH2840, significantly decreased the interaction between RelA/p65 and MSK1 (Fig. 6*B*).

We also tested whether OGG1 plays a role in the DNA occupation of MSK1, which could lead to phosphorylation of RelA/p65 at Ser276. Cells were transfected with siRNA-targeting *OGG1* or control, then exposed to TNFα for 1 h. Subsequently, DNAs were ChIP-ed with Abs recognizing OGG1, phospho-Ser276 RelA/p65, and MSK1. The chromatin immunoprecipitation (ChIP) assay revealed that OGG1 silencing (Fig. 6*C*) lowered the enrichment of MSK1 and phospho-Ser276 RelA/p65 to the promoters of group I genes (Fig. 6, *D* and *E*), consistent with the observed expression patterns of group I genes. However, OGG1 did not influence the enrichment of MSK1 and phospho-Ser276 RelA/p65 in group II gene promoters (Fig. 6, *D* and *E*). To further verify the function of OGG1, cells were treated with the antioxidants NAC or the OGG1 inhibitor TH5487, followed by TNFα incubation. The ChIP assay indicated that TH5487 or NAC significantly decreased the enrichment of phospho-Ser276 RelA/p65 and MSK1 on the promoters of group I, but not group II genes (Fig. S6, *A*–*C*).

**Figure 7:**
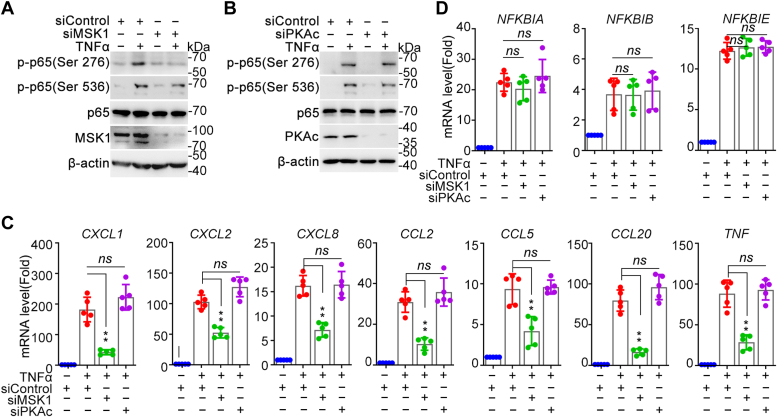
**MSK1 phosphorylates RelA/p65 at Ser276 and participates in selective gene regulation.***A* and *B*, MSK1, but not PKAc, mediates phosphorylation of RelA/p65 at Ser276. HEK293 cells were transfected with siRNA targeting MSK1 (*A*) or PKAc (*B*) and then were exposed to TNFα for 1 h. Phosphorylation of RelA/p65 at Ser276 and Ser536 were detected by Western blot. *C* and *D*, MSK1, but not PKAc, selectively regulates NF-κB–driven gene expression. HEK293 cells were transfected with short-interfering RNA targeting MSK1 or PKAc and then were exposed to TNFα for 1 h. The mRNA levels of *CXCL1*, *CXCL2*, *CXCL8*, *CCL2*, *CCL5*, *CCL20*, *TNF* (*C*) as well as *NFKBIA*, *NFKBIB*, *NFKBIE* (*D*) genes were assessed by qPCR. Data are expressed as mean ± SD. ∗∗*p* < 0.01, ns, not significant. MSK1, mitogen-and stress-activated kinase 1; qPCR, quantitative PCR.

Next, to further validate the impact of MSK1 on gene transcription efficiency, cells were transfected with siRNA targeting *MSK1* or control, followed by TNFα incubation. Western blotting revealed significant decrease in phospho-Ser276 RelA/p65 after MSK1 silencing with no effect on phosphor-Ser536 (Fig. 7*A*). In contrast, transfection of siRNA-targeting PKAc did not alter the level of phospho-Ser276 of RelA/p65 induced by TNFα stimulation (Fig. 7*B*). Subsequently, we assessed the effects of MSK1 or PKAc silencing on the expression of TNFα-stimulated NF-κB–driven genes. The results showed that the knockdown of MSK1 significantly inhibited the expression of group I genes without affecting that of group II genes (Fig. 7, *C* and *D*), consistent with the effect of OGG1 knockdown. PKAc knockdown did not impact the expression of any quantified genes (Fig. 7, *C* and *D*). Collectively, these data suggested that OGG1 is essential for the interaction between MSK1 and RelA/p65 as well as for the phosphorylation of RelA/p65 at S276 and facilitates the selective expression of NF-κB–driven genes.

### OGG1 augments recruitment of RelA/p65 and phosphorylated RNA polymerase II to promoter regions of pro-inflammatory cytokines and chemokines

**Figure 8:**
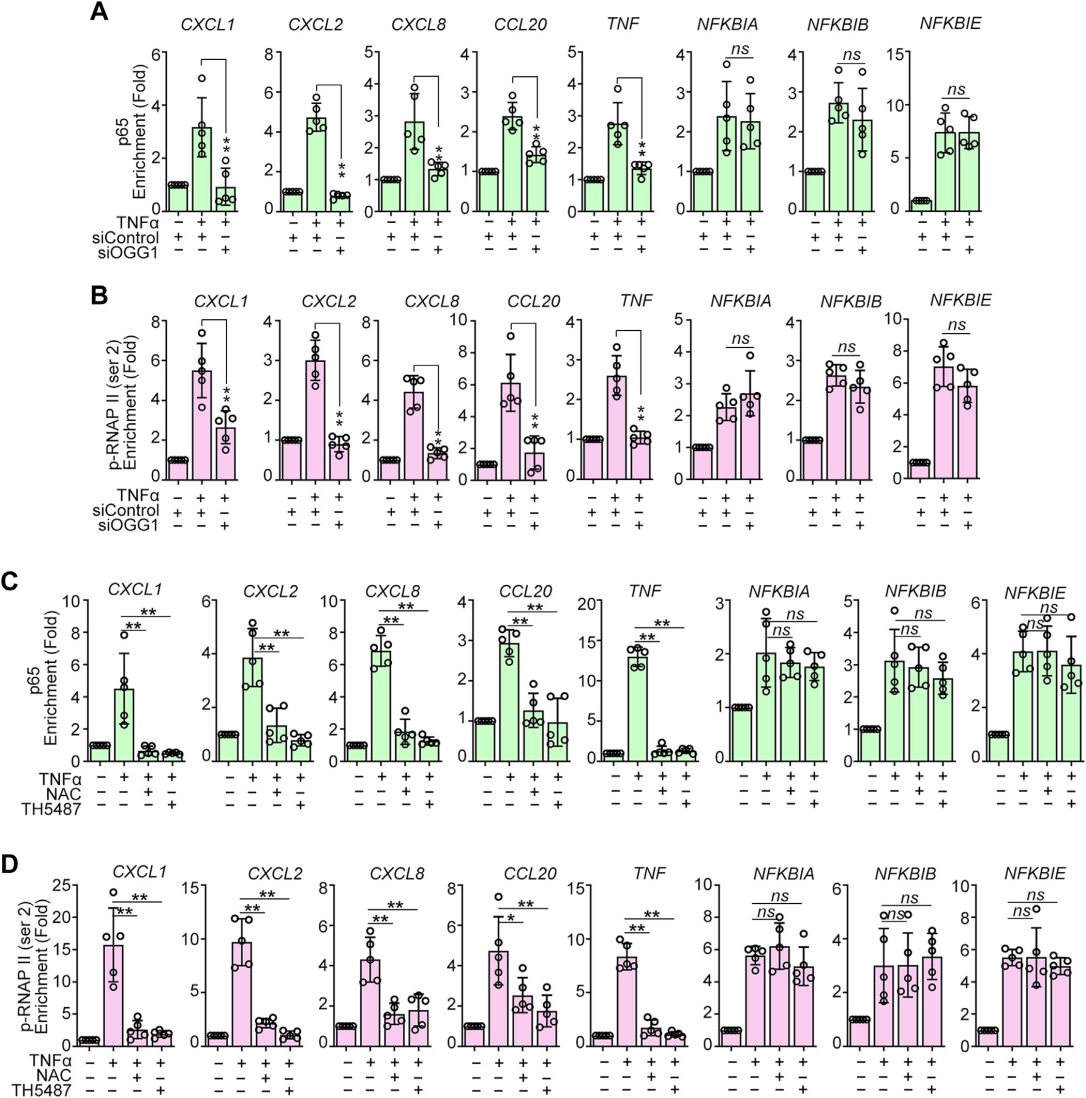
**OGG1 enhances recruitment of RelA/p65 and p-RNAP II to promoter.** HEK293 cells were transfected with siRNA targeting OGG1 or control for 36 h and then incubated with TNFα for 1 h (*A* and *B*) or transfected with Flag-OGG1 for 36 h and then incubated with TNFα for 1 h with or without pretreatment of NAC or TH5487 (*C* and *D*). Chromatin was isolated, and DNA was immunoprecipitated with Abs against RelA/p65 (*A* and *C*) and p-RNAP II (Ser2) (*B* and *D*) to assess the enrichment of RelA/p65 and p-RNAP II in promoter regions of pro-inflammatory cytokine/chemokine genes. The result of ChIP analysis was determined by qPCR. All experiments were performed five times. Data are expressed as mean ± SD. ∗*p* < 0.05, ∗∗*p* < 0.01, ns, not significant. NAC, N-acetyl-L-cysteine; OGG1, 8-oxoguanine DNA glycosylase 1; p-RNAP II, phosphorylated RNA polymerase II; qPCR, quantitative PCR.

Studies have addressed that the association between the site-specific RelA/p65 phosphorylation and the NF-κB activity with specific gene subsets is reflected in the levels of RelA/p65, RNA polymerase II (RNAP II), and phosphorylated RNA polymerase II (p-RNAP II) bound to their respective gene promoter regions (16, 17). In this study, we investigated whether OGG1 influences the DNA-binding properties of RelA/p65, RNA Pol II, and p-RNAP II. The results demonstrated that TNFα stimulation led to a significant increase in RelA/p65 and p-RNAP II enrichment on the promoters of all test genes. However, this enrichment was diminished by OGG1 knockdown specifically on the promoters of group I genes, while it had no effect on the promoters of group II genes (Fig. 8, *A* and *B*). Similarly, treatment with TH5487 or NAC significantly decreased TNFα-induced enrichment of RelA/p65 and p-RNAP II on the promoters of group I, but not group II genes (Fig. 8, *C* and *D*). These results were consistent with the observation that OGG1 knockdown/inhibition or ROS scavenging did not influence the transcription of group II genes (Figs. 2, *C*–*E* and S2, *B* and *C*).

Interestingly, there was a discrepancy in promoter enrichment of RNAP II between group I and group II genes in response to TNFα stimulation. While TNFα failed to induce a significant increase in RNAP II binding to promoters of group II genes (Fig. S7, *A* and *B*), the expression of group II genes was significantly upregulated (Figs. 1, *H*–*J* and S1, *A* and *B*). This may be attributed to the high basal preloading of RNAP II onto group II gene promoters as documented in previous studies (22). Taken together, these combined results suggested that group I genes require OGG1 for the recruitment of NF-κB and MSK1. Subsequently, MSK1 phosphorylates RelA/p65, facilitating the loading of RNA Pol II as well as its phosphorylation. In contrast, group II genes are preloaded with RNA Pol II, potentially priming their promoters for the rapid recruitment of RelA/p65 and the formation of a preinitiation complex.

## Discussion

NF-κB plays a fundamental role in the inflammatory response and is pivotal for maintaining immune system homeostasis. The phosphorylation of the NF-κB subunits is critical steps in its activation in response to various stimuli. Among the subunits, RelA/p65 has garnered significant attention. Phosphorylation of RelA/p65 at different sites forms a phosphorylation code, with Ser276 being one of the best-understood targets (14). While many kinases have been implicated in RelA/p65 Ser276 phosphorylation, the specificity of signaling induction and the identity of target genes remain unclear. In this study, we deciphered the underlying molecular mechanism how ROS-responsive RelA/p65 Ser276 phosphorylation is affected by OGG1. ROS attacks genomic DNA and produces 8-oxoGua primarily in the guanine-rich promoter regions. This substrate-bound OGG1 acts as a bridge between NF-κB and MSK1, which facilitates the phosphorylation of RelA/p65 at Ser276, thereby regulating the expression of ROS-sensitive pro-inflammatory cytokines and chemokines.

The transcriptional regulation of many, if not all, pro-inflammatory genes is regulated by ROS-mediated signaling (32, 33, 34). Treatment with ROS scavengers and antioxidants disrupted TNFα-induced phosphorylation of RelA/p65 at Ser276, leading to decrease in expression of *CXCL8* and *CXCL2*, while *NFKBIA* remained unaffected (22). Similar outcomes were observed when antioxidants were used to scavenge ROS induced by respiratory syncytial virus infection (12). Of note, TNFα-induced ROS signaling is not required for the classical IKK–NF-κB/RelA activation pathway, indicating there is a novel secondary signaling pathway triggered by ROS, controlling NF-κB activation, and critical in TNFα-induced gene expression (22). ROS promoted the enrichment of OGG1 at specific sites of the chromatin and recruited NF-κB to facilitate transcription of a subset of NF-κB targets. Depletion of OGG1 decreased DNA enrichment of NF-κB at *LTA*, *IL6*, *CCL20*, *TNF*, *CXCL1*, and *CXCL6* promoters but had no significant effects on the association of NF-κB with *RELA*, *NFKBIB*, and *ICAM1* promoters (9). Studies have also documented that RelA/p65 phospho-Ser276 is an important step in NF-κB regulation of a subset of genes, including *Cxcl2*, *Cxcl1*, *Ccl20*, etc., but not necessary for other genes such as *Nfkbia*, *Nfkbie*, and *Cxcl10* (16, 17). The combined data implicitly reveal a regulatory pathway: 8-oxoGua generated at promoter regions of specific ROS-responding genes may function as an epigenetic-like mark, and OGG1, as the cognate reader, binds to this substrate, recruits NF-κB, and promotes phosphorylation of RelA/p65 at Ser276.

Numerous protein kinases, including PKAc, MSK1, MSK2, PKC, Pim1, and RSK p90, participate in the phosphorylation of RelA/p65 at Ser276 under different conditions (14). Among these kinases, MSK1 and PKAc have been shown to phosphorylate RelA/p65 in a ROS-dependent manner as ROS scavengers can inhibit their activation (22, 24, 25). In addition, MSK1-induced Ser276 phosphorylation of RelA/p65 is activated by a number of inflammatory stimuli including IL-1, TNFα, and respiratory syncytial virus. It has been shown to promote the expression of *SCF*, *CXCL2*, and *IL-8* during inflammation (12, 35). In the present study, exposure to TNFα only induced the interaction between RelA/p65 and MSK1, but not with other kinases (Fig. 5, *A*–*E*). Furthermore, the interaction of RelA/p65 with MSK1 was disrupted by OGG1 knockdown or inhibition of substrate binding of OGG1 (Fig. 6, *A* and *B*). Ser276 of RelA/p65 has also been well characterized as a PKAc phosphorylation site. In resting cells, inactive PKAc is bound to cytosolic IκB:NF-κB complexes. Upon activation of the IKK complex and the subsequent degradation of IκB, active PKAc is liberated in response to LPS, TNFα, or TGF-β, leading to phosphorylation of RelA/p65 at Ser276 in a cAMP-independent process (36, 37, 38). Furthermore, PKAc phosphorylates RelA/p65 in the cytosol, while MSK1-mediated phosphorylation occurs in the nucleus (30, 39). This provides a context where OGG1 acts as a bridge, facilitating the interaction between RelA/p65 and MSK1. In contrast, phosphorylation of RelA/p65 on Ser536 constitutively occurs in the cytoplasm of both Jurkat or human peripheral blood T cells. This type of RelA/p65 phosphorylation did not occur in the canonical NF-κB complex of p50 and p65 (40). Likewise, TNFα stimulation induced ζPKC-mediated Ser311 phosphorylation of RelA/p65. Despite the impairment in κB-dependent transcription, RelA/p65 S311A nuclear translocation and interaction with the κB oligonucleotide probe were not affected (41). Hence, mainly nucleus-located OGG1 does not impact the level of phosphorylation of RelA/p65 at Ser536 or Ser311.

A previous study determined that the promoter of *NFKBIA* is pre-occupied with RNAP II, and the transcription of this gene occurs independently of RelA/p65 phospho-Ser276 regulation. Recruitment of RNAP II to the promoters of *CXCL8* and *CXCL2* is induced in response to TNFα stimulation, and transcriptional activation of these genes is regulated by Ser276 phosphorylation of RelA/p65 (22). Likewise, an extensive study involving an array of NF-κB–dependent genes revealed that phosphorylation-dependent changes in gene expression correspond to the level of RelA/p65 and p-RNAP II bound to the respective gene’s promoter regions. It was determined that Ser276 phosphorylation of RelA/p65 is essential for the recruitment of RNA Pol II and p-RNAP II to promoter sites of genes including *Cxcl2*, *Cxcl1*, *Ccl20*, etc. In contrast, RNA Pol II is associated with promoter sites of *Nfkbie* and *Cxcl10* without signal induction and RelA/p65 activation. Therefore, Ser276 phosphorylation of RelA/p65 is dispensable for upregulation of these genes (17). Furthermore, studies have revealed that RelA/p65 with phospho-Ser276 binds with the P-TEFb (containing cyclin T1 and CDK9), an elongation factor for RNAP II–directed transcription to phosphorylate the C-terminal domain of the largest subunit of RNAP II. This complex is required for *CXCL2* and *CXCL8*, but not for *NFKBIA* transcription activation (16, 42). Despite the discrepancy in the regulation of RNAP II loading by phospho-Ser276 RelA/p65, the role of OGG1 in MSK1-mediated RelA/p65 Ser276 phosphorylation, subsequently activating the transcription of a specific set of genes remains unknown.

Upon release from IκB in the cytoplasm, NF-κB translocates into the nucleus and induces expression of IκB, which constitutes a negative feedback loop in the core network module. Newly synthesized IκB binds to nuclear NF-κB and promoting its export from the nucleus to the cytoplasm. This drives oscillations in NF-κB translocation (43). Apparently, preloaded RNAP II on IκB promoter insures a timely establishment of negative feedback in response to activation of the canonical NF-κB pathway, regardless of the properties of the triggering signals. In this scenario, the allosteric changes introduced by DNA-bound RNAP II to the adjacent κB sites may create a relatively open interface for the rapid homing of NF-κB without the need for distinct phosphorylation modification of RelA/p65. Otherwise, responsive recruitment of NF-κB and the phosphorylation of NF-κB at different sites, which changes its affinity for coactivators such as CREB-binding protein and p300, are prerequisite for RNAP II to assume its role (44).

In the present study, expression of *NFKBIA*, *NFKBIB*, and *NFKBIE* (genes in group II) was not affected by OGG1, MSK1, and Ser276 phosphorylation of RelA/p65 (Figs. 2, *B*–*E*, 3, *B* and *C*, and 7, *C* and *D*). In contrast, *CXCL1*, *CXCL2*, *CXCL8*, *TNF*, etc. (genes in group I) are typically ROS-responding and immediately activated in the innate immune response. Their transcription shows a rapid and pronounced upregulation followed by a marked decrease. Importantly, the transcriptional kinetics of these pro-inflammatory cytokines/chemokines aligns with the levels of 8-oxoGua accumulation in the promoter regions (8, 26). While OGG1 binds with promoter regions of both groups of genes (Fig. 6*C*), the allosteric alterations in flanking regions introduced by OGG1–DNA interaction were necessary for NF-κB homing to the promoters of group I genes, but not group II genes. Additionally, DNA-bound OGG1 facilitates the interaction between MSK1 and RelA/p65, leading to the phosphorylation of RelA/p65 at Ser276. This benefits transcriptional activation of group I, but not group II, genes (Figs. 6 and 7).

DNA-binding sites themselves can also function as allosteric regulators of transcription (45). Studies have documented that phosphorylation of RelA/p65 at the differential serine residues modulates its transcriptional activity in a *cis*-acting element– and promoter-specific manner, leading to a gene expression profile dependent on phosphorylation state. The effects of RelA/p65 mutants on specific genes were examined using isolated linear κB sequences that do not resemble the complex nature of endogenous gene promoters; it is unlikely that histone-related promoter remodeling or interaction with other transcription factor is involved (46). Similarly, another study showed that S80 phosphorylation regulates the binding of p50 to NF-κB binding (κB) sites in a sequence-specific manner (47). Additionally, a study summarized that κB-binding sites in the promoters of genes dependent on Ser276 phosphorylation of RelA/p65 exhibited a preference for thymidine or maximum of three guanines at the 5′ end. The highest conservation was observed in genes that do not require Ser276 phosphorylation of RelA/p65 for their transcriptional activation, which displayed four to five guanine residues at the 5′ end of κB sites (17). Therefore, we speculate that while more guanine in the promoters of the groups II genes is beneficial for NF-κB recognition and binding to the κB site, the engagement of OGG1 with 8-oxoGua situated in the promoter not only facilitates homing of NF-κB to its sites but also mediates the interaction between MSK1 and RelA/p65. This leads to the phosphorylation of RelA/p65 at S276, thereby intensifying the affinity of NF-κB for the κB sites.

Taken together, our data underscore the importance of OGG1 in affecting the Ser276 phosphorylation of RelA/p65 for selective regulation of the transcription of the ROS-responding pro-inflammatory cytokine/chemokine genes. This study not only provides a new insight into the regulatory mechanisms of the phosphorylation codes of RelA/p65 but also reinforces the notion of a precise and intricate control of promoter-bound OGG1, that has evolved in aerobic cells to meet the need for timely gene transcription activation.

## Experimental procedures

### Antibodies and reagents

The polyclonal antibody anti-phospho-NF-kappaB p65 (pSer^276^) (Cat# SAB4504488) and monoclonal antibody against FLAG (Cat# F1804) were purchased from Sigma Aldrich. The monoclonal antibody against OGG1 (Cat# ab124741) was purchased from Abcam. The monoclonal antibody against Phospho-NF-κB p65 (Ser536) (Cat# 3033), Phospho-Rpb1 CTD (Ser2) (Cat# 13499), and MSK1 (Cat# C27B2) were purchased from Cell Signaling Technology. The monoclonal antibody against p65 (Cat# sc-8008X), PKAα cat (Cat# sc-28315), PKC α (Cat# sc-8393), and Pim-1 (Cat# sc-13513) were purchased from Santa Cruz. The monoclonal antibody against RNA pol II CTD (Cat#49-1033) was purchased from Thermo Fisher Scientific. The monoclonal antibody against GST (Cat# HT601), GAPDH (Cat# HC301), β-Tublin (Cat# HT101), LaminA/C (Cat# HA105), GFP (Cat# HT801), and β-actin (Cat# HT201) were purchased from TRANS.

### Cell culture and treatment

WT, *Ogg1*^*−/−*^, *RelA*/*p65*^*−/−*^ MEF cells, and HEK293 cells were grown in Dulbecco’s modified Eagle’s medium (Gibco) supplemented with 10% fetal bovine serum. Cells were treated with or without IKK inhibitor BMS-345541 (Sigma, Cat# B9935), 10 mM of NAC (Sigma, Cat# A7250), 10 μM of OGG1 inhibitor TH5487 (ProbeChem, Cat#, PC-35806),7,8-Dihydro-8-oxoguaninetriphosphatase inhibitor TH588 (Sigma, Cat# 5309160001), TH2840 (Provided by Dr T. Helleday, Karolinska Institute, Stockholm, Sweden;) for 1 h, followed by exposure to 20 ng/ml TNFα (Sigma, Cat# H8961) for various durations of time.

### Constructs and transfection

The eukaryotic expression plasmids Flag-OGG1 were constructed by inserting the coding sequence of OGG1 into pcDNA3.1 (+) and pZsYellow1-C1 vectors, respectively. Restriction enzymes EcoR I and Hind III were selected for cloning of pcDNA3.1 (+), and Hind III and BamH I were chosen for pZsYellow1-C1. GFP-RelA/p65 was produced by inserting the p65 coding sequence into PEGFP-N1 using restriction enzymes Hind III and Kpn I. GFP-RelA/p65 S276A, GFP-RelA/p65 S311A, and GFP-RelA/p65 S536A were created using the Fast Mutagenesis System based on GFP-RelA/p65. Flag-MSK1 and Flag-PKAc were constructed by inserting the coding sequence of MSK1 and PKAc into p3× FLAG-CMV-9, respectively. Restriction enzymes EcoR I and Kpn I were selected for cloning of Flag-MSK1, and Hind III and Kpn I were chosen for Flag-PKAc, respectively. All sequence-verified plasmids were transfected using Lipofectamine 3000 reagent (Invitrogen) according to the manufacturer's instructions. Cells were treated with or without TNFα after 36 h of transfection. The prokaryotic expression plasmids GST-MSK1 and GST-PKAc were produced by inserting the MSK1 and PKAc coding sequence into pGEX-4T-2 using restriction enzymes Sma I and Not I.

### Down regulation of gene expression

HEK293 cells were transfected with siRNA targeting hOGG1, hMSK1, hPKAc mRNAs, or with universal siRNA negative control using Lipofectamine 3000 reagent. The siRNA-SMARTpool to downregulate human OGG1 (Cat# L-005147-00-0020) was purchased from Dharmacon (9). The siRNA for human MSK1 and PKAc knockdown was ordered from GENEWIZ. The sequences are siMSK1: 5′-GCAUGAGGUGCAGAUUUA-3′ and siPKAc: 5′-UCCUCUGGUAGAUGGCAAUCCAGUC-3’. The efficiency of downregulation was determined by qPCR and Western blot analysis.

### Quantitative PCR

Total RNA was obtained from cultured cells using TRIzol reagent (TIANGEN, Cat# DP421). One microgram-purified RNA from each sample was reverse-transcribed to the complementary DNA using PrimeScript RT reagent Kit (TaKaRa, Cat# RR037A). Then, the cDNA was used as template for qPCR. qPCR was accomplished by using the SYBR Green qPCR Master Mix (Cat# 638320, TaKaLa, Cat# RR420A). The relative levels of each sample were normalized to the housekeeping gene Gapdh, and expression of target genes was calculated using the ΔΔ Ct method. Primer sequences are shown in Table 1.

**Table 1:** Primer sequences for PCR

*GAPDH*	F: 5′-ACATCGCTCAGACACCATG-3′ R: 5′-TGTAGTTGAGGTCAATGAAGGG-3′
*CXCL1*	F: 5′-TCTCTCTTTCCTCTTCTGTTCCTA-3′ R: 5′-CATCCCCCATAGTTAAGAAAATCATC-3′
*CXCL2*	F: 5′-TGTTTGAGCATCACTTAGGAGAA-3′ R: 5′-CCCTGCCGTCACATTGATCT-3′
*CXCL8*	F: 5′-CCAGGAAGAAACCACCGGAA-3′ R: 5′-ATGAATTCTCAGCCCTCTTCAA-3’;
*CCL2*	F: 5′-AGCAGCCACCTTCATTCC-3′ R: 5′-GCCTCTGCACTGAGATCT TC-3′
*CCL5*	F: 5′-TCCTCATTGCTACTGCCCTC-3′ R: 5′-TCGGGTGACAAAGACGACTG-3′
*CCL20*	F: 5′-CCATGTGCTGTACCAAGAGT-3′ R: 5′-TTAGGATGAAGAATACGGTCTGTG-3′
*TNF*	F: 5′-TCAGCTTGAGGGTTTGCTAC-3′ R: 5′-TGCACTTTGGAGTGATCGG-3′
*NFKBIA*	F: 5′-CTCCGAGACTTTCGAGGAAATAC-3′ R: 5′-GCCATTGTAGTTGGTAGCCTTCA-3′
*NFKBIB*	F: 5′-GCCGACGCAGATGAATGG-3′ R: 5′-CCTCAGTGACGTAGCCGAAGA-3′
*NFKBIE*	F: 5′-ACCCGTCAAGGAACCACA-3′ R: 5′-CCGTCCTCGGAGATGTAAGTG-3′
*OGG1*	F: 5′-CAGAAGATAAGAGGACGCAGAAG-3′ R: 5′-CATATGAGGAGGCCCACAAG-3′
*Gapdh*	F: 5′-CTCATGACCACAGTCCATGC-3′ R: 5′-CACATTGGGGGTAGGAACAC-3′
*Cxcl1*	F: 5′-TCCAGAGCTTGAAGGTGTTGCC -3′ R: 5′- AACCAAGGGAGCTTCAGGGTCA-3′
*Cxcl2*	F: 5′-CAGAAGTCATAGCCACTCTCAAG-3′ R: 5′-GTGGAGTCATACTGGAACATGTAG-3′
*Ccl2*	F: 5′-CATCCACGTGTTGGCTCA-3′ R: 5′-AACTACAGCTTCTTT GGG ACA-3′
*Ccl5*	F: 5′-ACTCCCTGCTGCTTTGCCTAC-3′ R: 5′-GGCGGTTCCTTCGAGTGACAA-3′
*Ccl20*	F: 5′-CCAGCACTGAGTACATCAACT-3′ R: 5′-GTATGTACGAGAGGCAACAGTC-3′
*Tnf*	F: 5′-AGACCCTCACACTCAGAT CA-3′ R: 5′-TCTTTGAGATCCATGCCGTTG-3′
*Nfkbia*	F: 5′-TCCTGCACTTGGCAATCATC-3′ R: 5′-AGCCAGCTCTCAGAAGTGCC-3′
*Nfkbib*	F: 5′-TGCCTCAGATACCTACCTCACT-3′ R: 5′-GCTTCTAGTTGTAGCCTCCAGT-3′
*Nfkbie*	F: 5′-GGGCACGAGTGGAAAGAC-3′ R: 5′-GGATGAGATGCTGTTGAGGC-3′

### Co-IP and western blotting

Parallel cultures of YFP-OGG1–expressing cells (1 × 10^7^ HEK293) were stimulated with TNFα for 1 h and cells were lysed using buffer (Cell Signaling Technology; Cat# 9803; 50 mM Tris–HCl, pH 7.5, 150 mM NaCl, 1 mM EDTA, 1 mM EGTA, 1% Nonidet P-40) containing 2.5 mM sodium pyrophosphate, 1 mM glycerophosphate, and protease inhibitors (1 mM Na_3_VO_4_, 1 mM NaF, and 20 μg/ml aprotinin/leupeptin/phenylmethanesulfonyl fluoride). Next, the cell lysates were clarified (14,000*g* at 4 °C for 30 min) and then incubated with GFP beads (CH10001) for 3 h at 4 °C with rotation. Beads were washed three times and the proteins were eluted by boiling in 1×loading buffer for Western blot. Proteins were separated by 10% of SDS-PAGE and transferred onto nitrocellulose membranes. Membranes were blocked with 5% skim milk (in 0.1% TBST) for 1h and incubated with primary antibodies overnight at 4 °C. After three washes with TBST, membranes were incubated with secondary antibodies (LF101, LF102) and detected by ECL (Tanon) Plus Western blot detection reagents.

### Immunofluorescence

HeLa or A549 cells were plated on collagen pretreated cover glasses and transfected with GFP, GFP-RelA/p65, GFP-RelA/p65 S276A, GFP-RelA/p65 S311A, or GFP-RelA/p65 S536A. After TNFα treatment, cells were fixed with 4% paraformaldehyde (Dingguo, Cat# AR0211) for 10 min at room temperature and then permeabilized with 0.5% (v/v) Triton X-100 for 10 min. Subsequently, cells were incubated with 10% fetal bovine serum for 1 h at room temperature, and p65 antibody was added to individual slides at a dilution recommended by the manufacturer (1–200) in PBS for overnight incubation at 4 °C. After washing cells in 0.1% (w/v) Triton X-100 diluted in PBS (PBST), a secondary antibody (Alexa Fluor 594 goat anti-mouse IgG, red fluorescent) was added. Following another wash in PBST, the cell nuclei were stained with 4,6-diamidino-2-phenylindole for 5 min. Cells were visualized using a confocal microscope (Nikon) equipped with a 60× oil-immersion objective lens.

### Proximity ligation assay

HeLa cells were plated on collagen-treated coverslip slides with or without NAC or TH5487 for 1 h, followed by exposed to TNFα for 1 h. Cells were fixed with 4% paraformaldehyde and permeabilized with PBST for 5 min. After washing in PBS, Duolink blocking solution was added and PLA was performed using the Duolink PLA kit (OLink Biosci-ence, Cat# LNK-92101-KI01) according to the manufacturer’s instructions as we described previously (48).

In brief, cells were incubated with the primary antibodies to OGG1 (Novus, NB100-106) and MSK1 (Immunoway, YT2902) for 1 h at 37 °C. Afterward, cells were washed twice with 1X wash buffer A (Millipore Sigma, Cat# DUO82049). Subsequently, secondary antibody–conjugated MINUS and PLUS probes were added for 1 h at 37 °C. Following another wash with 1× wash buffer A, ligation mix was added to each sample for 30 min at 37 °C, and amplification mix was added for 100 min at 37 °C. After washing with buffer B (Millipore Sigma, Item # DUO82049), cells were mounted with mounting medium (ibidi Inc, Cat# 19-06-14). Cells were visualized using a confocal microscope (Nikon) equipped with a 60× oil-immersion objective lens.

### GST-fused protein purification and pull down

GST and GST-fused proteins were induced in *Escherichia coli* BL21. Overexpression was achieved by adding 1 mM of IPTG and 40 μM of ZnSO_4_ to a culture with an OD of 1.0, and the cells were cultured at 16 °C overnight. After sonication, whole bacteria lysates were incubated with glutathione Sepharose 4B (GE Healthcare Life Science). GST-tagged proteins were purified following the manufacturer's instructions. Recombinant His-OGG1(Cat# NBP1-45318) was purchased from NOVUS. Subsequently, a GST pull-down experiment was conducted using BeyoGold GST-Tag Purification Resin (Beyotime, Cat# P2250) in accordance with the manufacturer's instructions. Eluted proteins were separated by SDS-PAGE electrophoresis, and Western blot analysis was performed.

### CHIP assay

Parallel 10-cm dish cultures of HEK293 cells were transfected with si*OGG1* for 48 h and then exposed to 20 ng/ml TNFα for 1 h. DNA–protein complexes were cross-linked with 1% paraformaldehyde for 10 min at room temperature and sheared by sonication with 30-s pulses (average 300 bp fragment) repeated six times on high power using a Bioruptor Plus from Diagenode. Approximately, 25 μg of genomic DNA fragments per sample were immunoprecipitated using 2 μg of antibody overnight at 4 °C and then incubated with 30 μl of magnetic beads (Bimake, Cat# B23202) for 3 h. The precipitates were washed three times, de-crosslinked, and subjected to qPCR following the manufacturer's instructions for the Simple ChIP Enzymatic Chromatin IP Kit (Cell Signaling Technology). Primers for qPCR amplification are provided in Table 2, and the size of the qPCR product is approximately 200 bp. ChIP-qPCR calculations were performed as previously described (9). In brief, the signal intensity value of DNA immunoprecipitated with the protein-specific antibody was divided by the intensity value of the IgG-immunoprecipitated signal and then normalized by the control, representing the fold enrichment of the protein on the specific region of genomic DNA.

**Table 2:** CHIP primer sequences for PCR

*CXCL1* (−208 to −9)	F: 5′-GGAGTTACTCTGAAGGGCGAG-3′ R: 5′- GAGATCCGCGAACCCCTTT -3′
*CXCL2* (−227 to −19)	F: 5′-ATTCGGGGCAGAAAGAGAAC-3′ R: 5′-ACCCCTTTTATGCATGGTTG-3′
*CXCL8* (−185 to +56)	F: 5′-AACTTTCGTCATACTCCG -3′ R: 5′-TTCTTCCTGGCTCTTGTC-3′
*CCL20* (−176 to −9)	F: 5′-ACCCTGACCTTCGCACCT-3′ R: 5′-CCTGGGATGGCCCTATTT-3′
*TNF* (−201 to −5)	F: 5'- CTTGTGTGTCCCCAACTT−3' R: 5′- GGGTGTGCCAACAACTGC−3′
*NFKBIA* (−154 to −15)	F:5′- GCTCAGGGTTTAGGCTTCT -3′ R: 5′-GGCACGGACTGCTGTGGG -3′
*NFKBIB* (−134 to +99)	F: 5′- GAGAGTTGTAGTCCTCCCGA −3′ R: 5′- GTCGGCAGCTTTTCCCAAG−3′
*NFKBIE* (−185 to −56)	F: 5′-GGGAACCACAGACTCCAAGC−3′ R: 5′-ACCAACAGGGTCGCCTCA−3′

### Statistical analysis

The data are expressed as the mean ± SD. Results were analyzed for significant differences using unpaired nonparametric analysis (Mann–Whitney test). Differences were considered significant at *p* < 0.05 (∗*p* < 0.05, ∗∗*p* < 0.01).

## Data availability

All data generated or analyzed during this study are included in this article or are available from the corresponding author (Xueqing Ba, E-mail: baxq755@nenu.edu.cn) upon reasonable request.

## Supporting information

This article contains supporting information.

## Conflict of interest

The authors declare that they have no conflicts of interest with the contents of this article.
