# Supplementary material for: 8-Oxoguanine DNA glycosylase 1 selectively modulates ROS-responsive NF-κB targets through recruitment of MSK1 and phosphorylation of RelA/p65 at Ser276
Source: J Biol Chem. 2023 Sep 29;299(11):105308. doi: 10.1016/j.jbc.2023.105308 (PMC10641171; doi:10.1016/j.jbc.2023.105308)
Supplement: Supporting information [file mmc1.docx]

***Supporting Information***

**8-Oxoguanine DNA glycosylase 1 selectively modulates ROS-responsive NF-κB targets through recruitment of MSK1 and phosphorylation of RelA/p65 at Ser276**

Yaoyao Xue^1, 2^, Chunshuang Li^1, 2^, Shihua Deng^1, 2^, Xin Chen^1, 2^, Jinling Han^1, 2^, Xu Zheng^1, 2^, Miaomiao Tian^1, 2^, Wenjing Hao^3^, Lang Pan^4^, Istvan Boldogh^4^, Xueqing Ba^1, 2,^ *, Ruoxi Wang^5,^ *

Running title: OGG1 recruits MSK1 to phosphorylate RelA/p65 Ser276

^1^ Key Laboratory of Molecular Epigenetics of Ministry of Education, Northeast Normal University, Changchun, Jilin, China

^2^ College of Life Sciences, Northeast Normal University, Changchun, Jilin, China

^3^ Institute of Biomedical Sciences, College of Life Sciences, Key Laboratory of Animal Resistance Biology of Shandong Province, Shandong Normal University, Jinan, Shandong, China

^4^ Institute of Genetics and Developmental Biology, Chinese Academy of Sciences, Beijing, China

^5^ Department of Microbiology and Immunology, University of Texas Medical Branch at Galveston, Galveston, USA

*: Correspondence to Ruoxi Wang: wangruoxi@sdnu.edu.cn and Xueqing Ba: baxq755@nenu.edu.cn

**
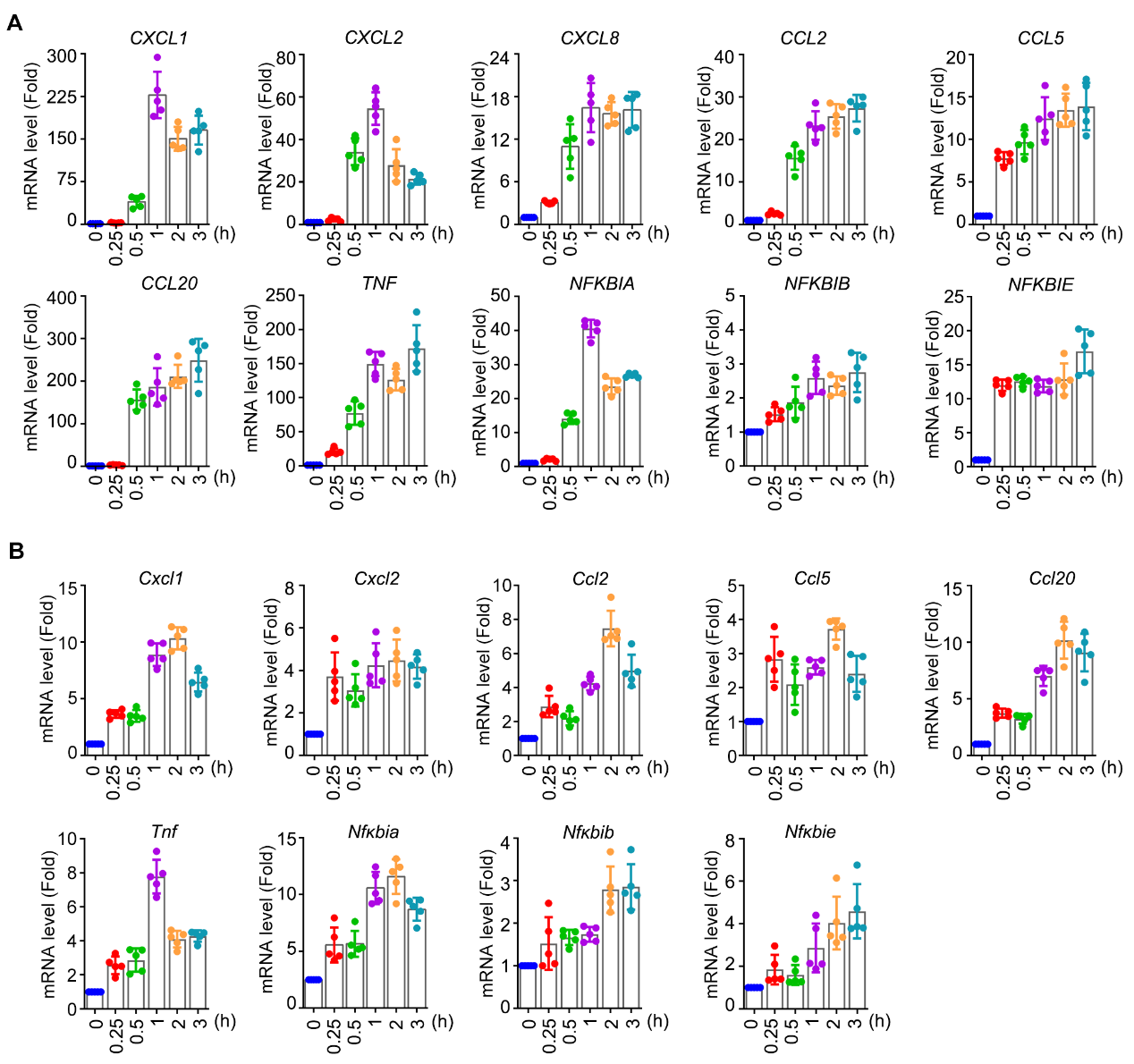
**

**Supplementary Figure 1. Time kinetics of the expression of inflammation-responding genes.** HEK293 cells (A) and MEF cells (B) were exposed to TNFα for 0, 0.25, 0.5, 1, 2, 3 h. The mRNA levels of *CXCL1*, *CXCL2*, *CXCL8*, *CCL2*, *CCL5*, *CCL20*, *TNF*, *NFKBIA*, *NFKBIB* and *NFKBIE* gene (A)and *Cxcl1*, *Cxcl2*, *Cxcl8*, *Ccl2*, *Ccl5*, *Ccl20*, *Tnf*, *Nfkbia*, *Nfkbib* and *Nfkbie* gene (B) were assessed by real-time qPCR. All experiments were performed five times.

**
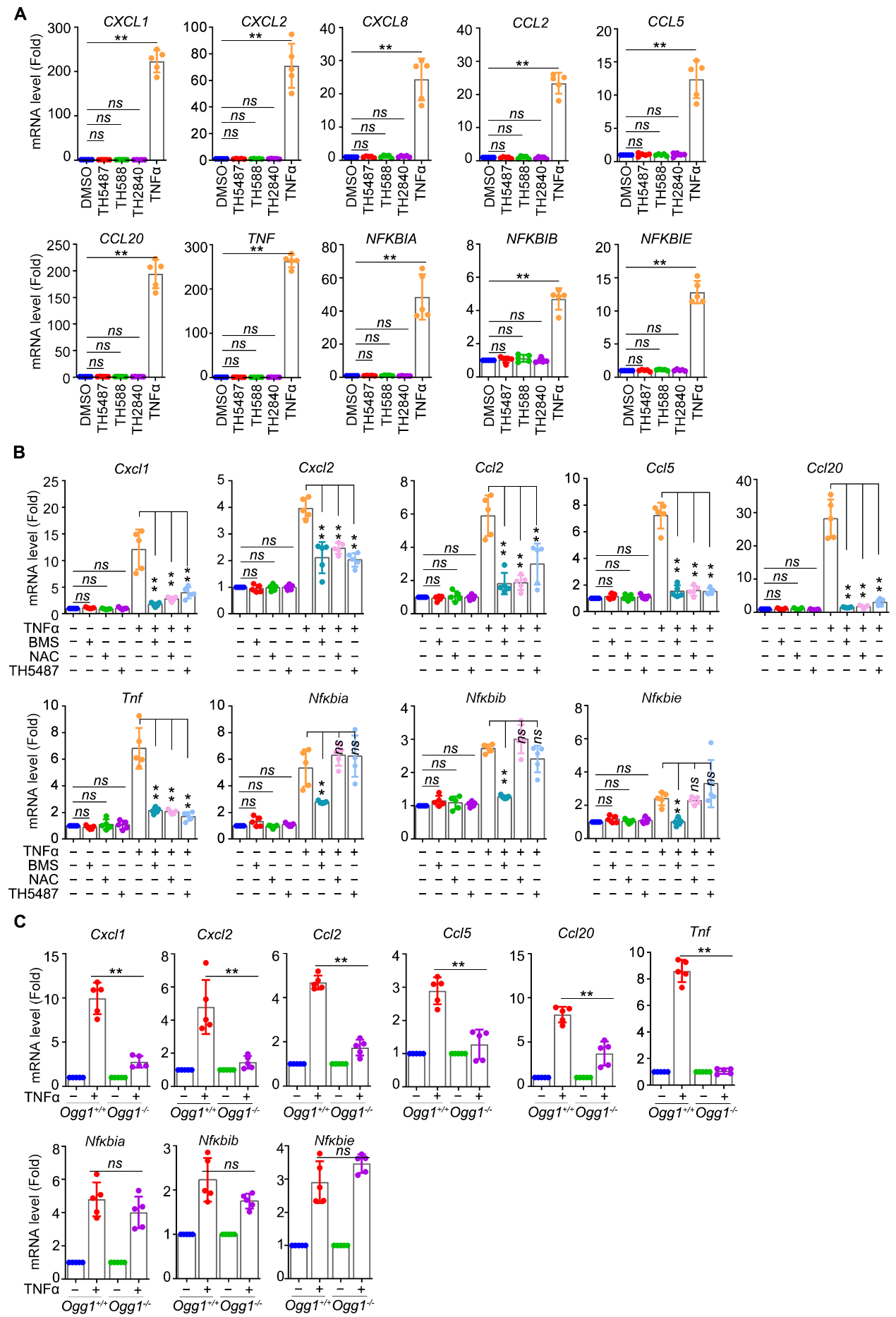
**

**Supplementary Figure 2. OGG1 and ROS signaling are required for expression of NF-κB-driven gene expression. (A)** TH5487, TH2840 or TH588 has no effect on gene expression without TNFα stimulation. HEK293 cells were treated with DMSO, TH5487, TH588 or TH2840 for 2 h, and the mRNA levels of *CXCL1*, *CXCL2*, *CXCL8*, *CCL2*, *CCL5*, *CCL20*, *TNF*, *NFKBIA*, *NFKBIB* and *NFKBIE* gene were assessed by real-time qPCR. (B and C) OGG1 and ROS signaling are required for expression of NF-κB-driven gene expression in MEF. MEF cells were exposed to TNFα for 1 h with or without pretreatment of BMS-345541, NAC or TH5487 (B) or *Ogg1^+/+^* or *Ogg1^−/−^* MEF cells were treated with TNFα for 1 h (C). *Cxcl1*, *Cxcl2*, *Ccl2*, *Ccl5*, *Ccl20*, *Tnf*, *Nfkbia*, *Nfkbib* and *Nfkbie* gene expression was assessed by real-time qPCR. All experiments were performed five times. Data are expressed as mean ± SD. **p < 0.01, ns, not significant.

**

**

**Supplementary Figure 3. ROS signaling is required for RelA/p65 phosphorylation.** (A) NAC, TH5487 or TH2840 has no effect on phospho-Ser276 RelA/p65 without TNFα stimulation. HEK293 cells or MEF cells were treated with NAC, TH5487 or TH2840 for 2 h, RelA/p65 S276 phosphorylation and S536 phosphorylation were detected by Western blot. (B) Kinetics change in phosphorylation of NF-κB/RelA at S276. MEF cells were exposed to TNFα for 0, 0.25, 0.5, 1, 2, 3h. RelA/p65 S276 phosphorylation and S536 phosphorylation were detected by Western blot. (C) RelA/p65 S276 phosphorylation is ROS signaling-responsive. MEF cells were exposed to TNFα for 1h with or without pretreatment of NAC. RelA/p65 S276 phosphorylation and S536 phosphorylation were detected by Western blot. (D) Efficacy of shOGG1. HeLa-shCTR or HeLa-shOGG1 cells and A549-shCTR or A549-shOGG1cells’ whole cell extracts were made and OGG1 expression was determined by Western blotting. (E) OGG1 has no role in translocation induction of RelA/p65. *Ogg1^+/+^* or *Ogg1^−/−^* MEF cells were treated with TNFα for 0, 0.25, 0.5, 1, 2, 3 h, and then, were fractionated into cytoplasmic extraction (CE) and nuclear extraction (NE) preparations for analysis by Western blotting using the indicated Abs All experiments were performed three times.


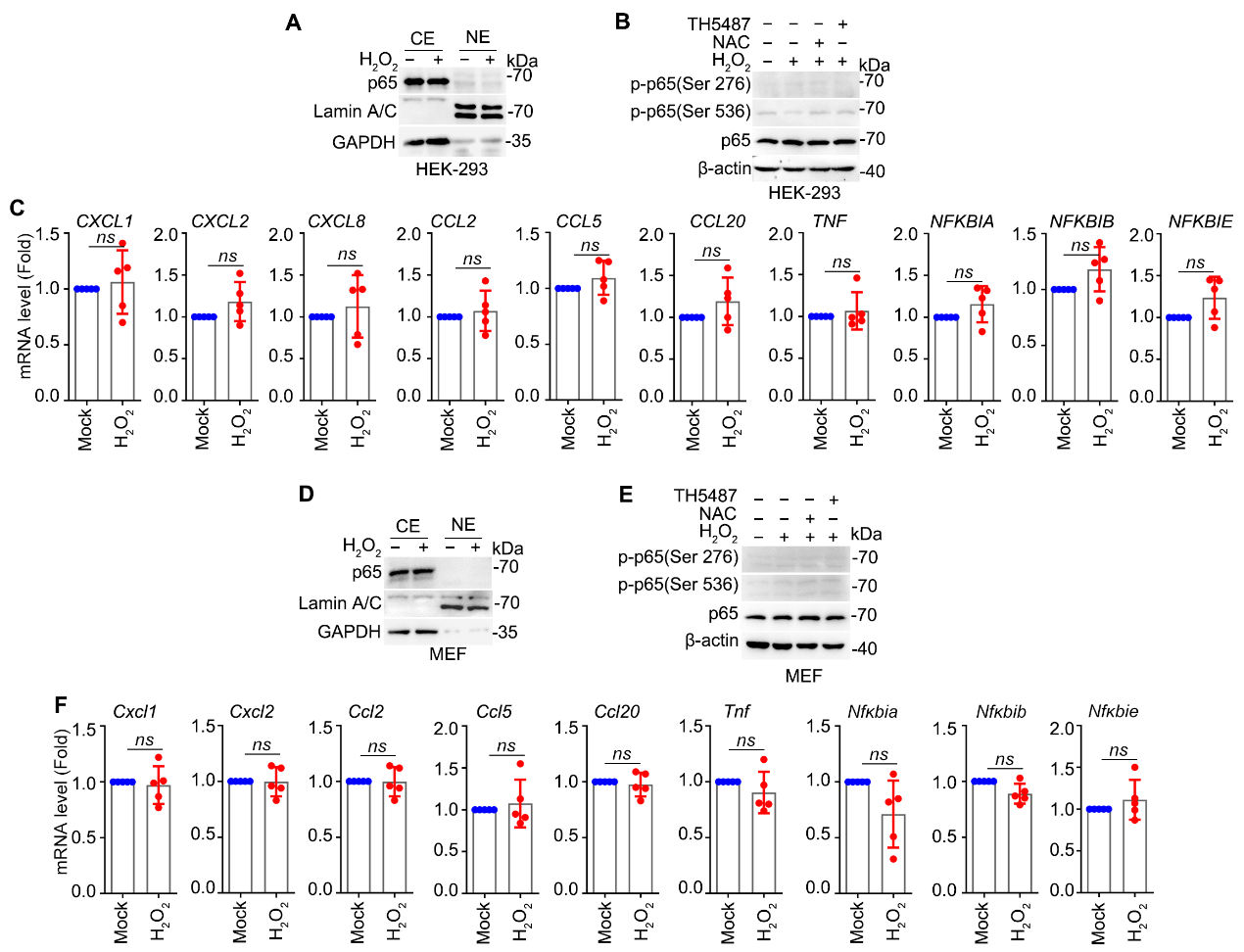


**Supplementary Figure 4. H_2_O_2_ itself does not activate NF-κB.** (A and D) H_2_O_2_ can’t induced RelA/p65 translocation into the nucleus. HEK293 cells (A) or MEF cells (D) were treated with H_2_O_2_ (200 μM) for 30 min, and then, were fractionated into cytoplasmic extraction (CE) and nuclear extraction (NE) preparations for analysis by Western blotting using the indicated Abs. (B and E) H_2_O_2_ can’t induced formation of phospho-Ser276 RelA/p65. HEK293 cells (B) or MEF cells (E) were exposed to H_2_O_2_ for 1h with or without pretreatment of NAC or TH5487 RelA/p65 S276 phosphorylation and S536 phosphorylation were detected by Western blot. (C and F) H_2_O_2_ incubation did not induce up-regulation of these inflammatory genes. HEK293 cells (C) or MEF cells (F) were exposed to H_2_O_2_ for 1h, these inflammatory gene expression was assessed by real-time qPCR. Data are expressed as mean ± SD. ns, not significant.

**
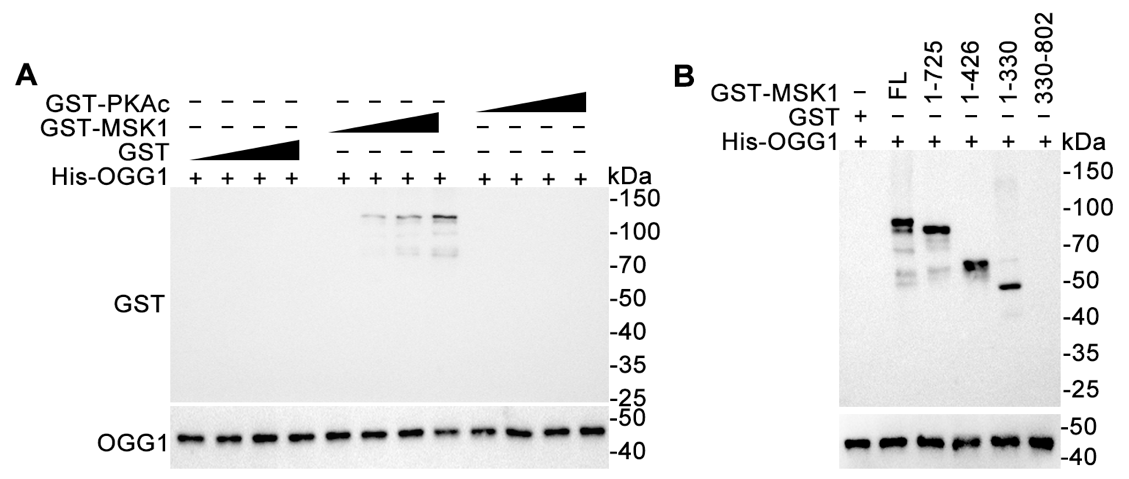
**

**Supplementary Figure 5. Physical interaction between OGG1 and MSK1.** (A) Interaction between OGG1 and MSK1 *in vitro*. Increasing concentration of GST, GST-MSK1 or GST-PKAc were incubated with His-OGG1 was performed in pull down assay. Levels of pulled-down OGG1 were detected by western blot. (B) NTKD mediates the association of OGG1 with MSK1. GST-MSK1 or deletion mutants were incubated with His-OGG1 and pull down assay was performed. A representative result of three experiments is shown. Levels of pulled-down OGG1 were detected by western blot.

**
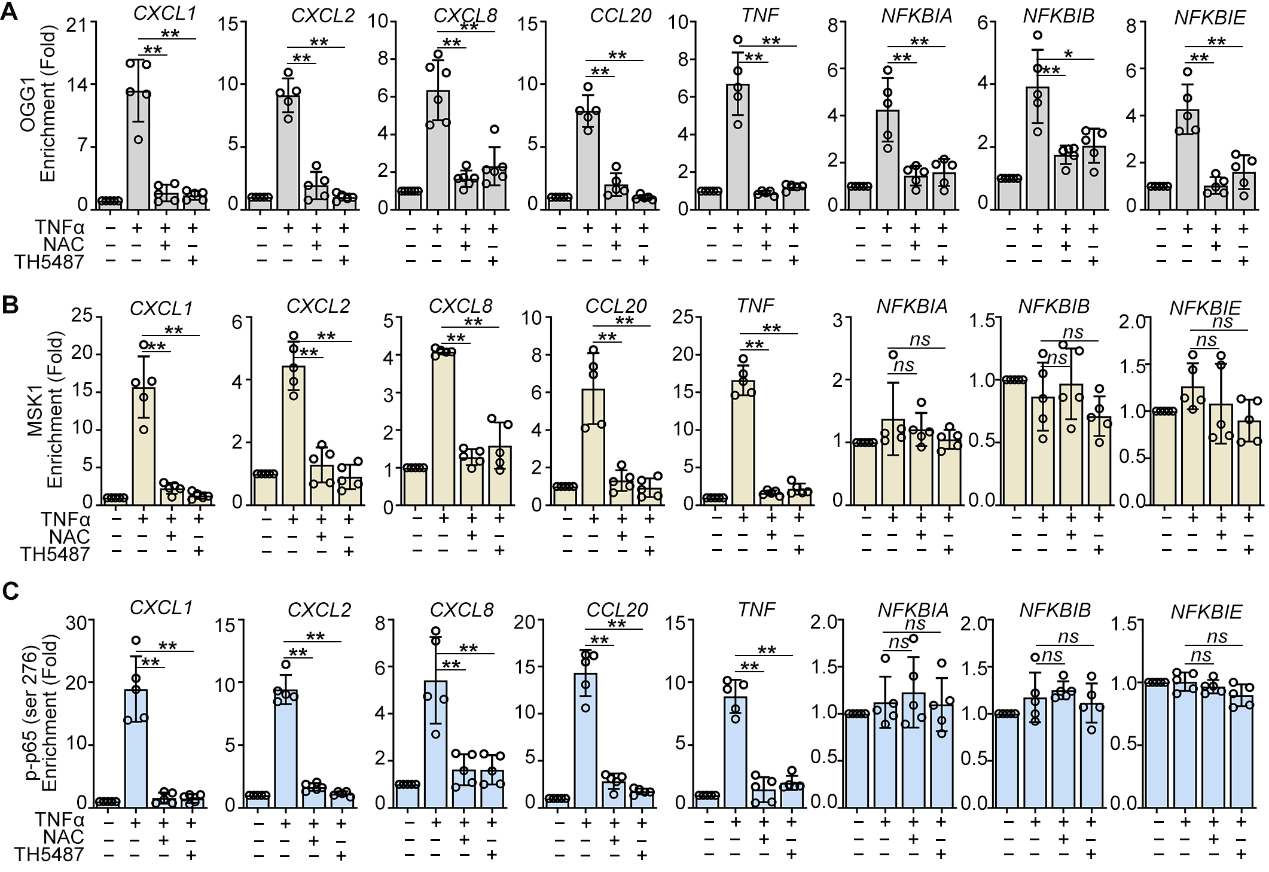
**

**Supplementary Figure 6. OGG1 facilitates the enrichment of MSK1 and p-RelA/p65 (Ser 276) in promoter regions of pro-inflammatory cytokine/chemokine genes.** (A-C) HEK293 cells were transfected with Flag-OGG1 for 36 h, and then incubated with TNFα for 1 h with or without pretreatment of NAC or TH5487. Chromatin was isolated, and DNA was immunoprecipitated with Flag (A), MSK1 (B) and p-p65 (Ser 276). ChIP analysis was carried out by real-time qPCR. All experiments were performed five times. Data are expressed as mean ± SD. *p < 0.05, **p < 0.01, ns, not significant.


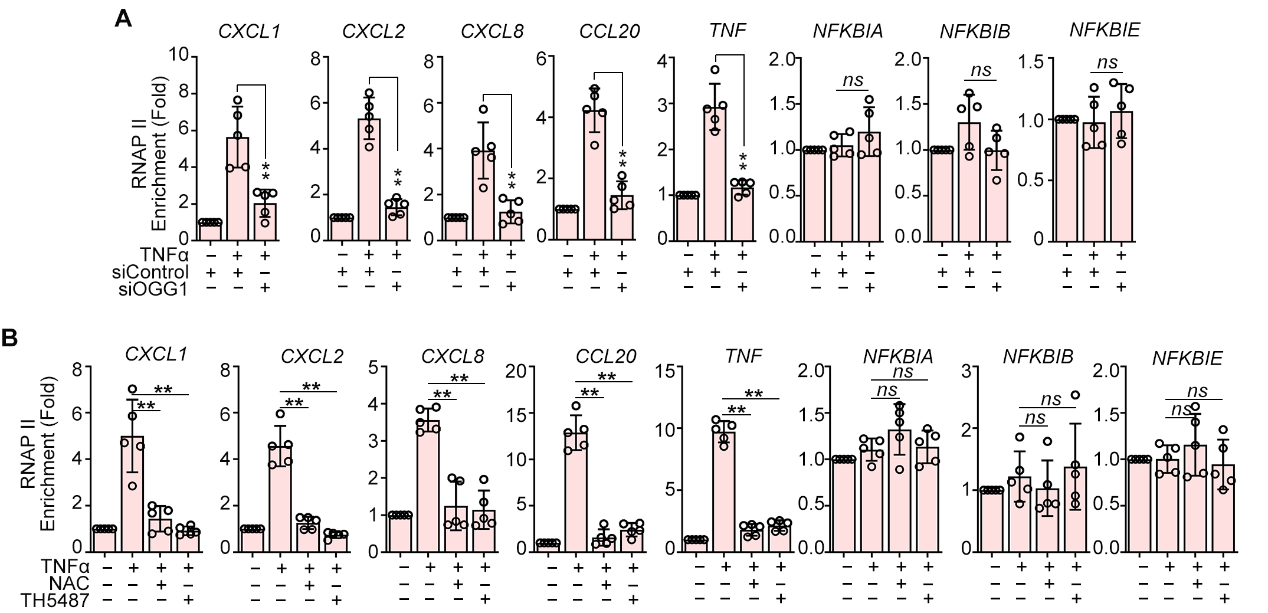


**Supplementary Figure 7. OGG1 facilitates recruitment of RNAP II to promoter regions of pro-inflammatory cytokines/chemokines.** HEK293 cells were transfected with siRNA targeting OGG1 or control for 36 h, and then incubated with TNF TNFα for 1 h (A) or were transfected with Flag-OGG1 for 36 h, and then incubated with TNFα for 1 h with or without pretreatment of NAC or TH5487 (B). Chromatin was isolated, and DNA was immunoprecipitated with Abs against RNAP II to detect the enrichment of RNAP II in promoter regions of pro-inflammatory cytokine/chemokine genes. The result of ChIP analysis was determined by real-time qPCR. All experiments were performed five times. Data are expressed as mean ± SD. **p < 0.01, ns, not significant.
